# Oilbirds disperse large seeds at longer distance than extinct megafauna

**DOI:** 10.1038/s41598-020-79280-4

**Published:** 2021-01-11

**Authors:** Pablo R. Stevenson, Laura Cardona, Sasha Cárdenas, Andrés Link

**Affiliations:** Laboratorio de Ecología de Bosques Tropicales Y Primatología, Departamento de Ciencias Biológicas, Universidad de Los Andes, Carrera 1 No. 18A-10, Bogotá, Colombia

**Keywords:** Ecology, Zoology

## Abstract

The extinction of megafauna in the Neotropics is thought to have reduced the potential of large seeds to be dispersed over long distances by endozoochory (ingestion by animals), but some seed dispersal systems have not been considered. We describe the role of oilbirds (*Steatornis caripensis*) as seed dispersers, in terms of seed width and dispersal distance (using GPS tracking devices), and we compare with data reported for other animals. Oilbirds dispersed seeds up to 29 mm wide, with a mean dispersal distance of 10.1 km (range 0–47.6 km). Some components of seed dispersal by oilbirds are outliers compared to that of other frugivores, such as the relationship between maximum seed width and body weight (however, few other extant specialized frugivores are also outliers). Estimates of mean dispersal distance by oilbirds are the largest reported, and we confirm that some living frugivores currently fulfil roles of seed dispersers and ecosystem services previously assumed to be only performed by extinct species.

## Introduction

Animals play key ecological roles as seed dispersers, and have the capacity to change the floristic composition and modify ecosystem functioning. For example, in areas where large frugivore primates are present, plant recruitment is well represented by the species they prefer and disperse by endozoochory (when seeds enter the digestive system of animals)^1,2^. In addition to the changes in composition, these studies have found higher plant diversity where these frugivores have large populations than in areas with small population size or where the frugivores have gone locally extinct. Furthermore, sites with the presence of efficient seed dispersers are expected to capture more carbon than defaunated areas^3–5^, potentially affecting global climate cycles.

Seed dispersal is important in allowing seeds to reach new establishment sites (especially under climate change scenarios)^6–8^, and because of negative density and distance dependent mortality of seeds and seedlings^9–12^. Also, long distance dispersal may affect the rates of population migration and contributes to maintaining connectivity among plant populations in fragmented landscapes and across large spatial scales^13,14^. Seed dispersal agents that travel long distances are especially important in sustaining the spatial and genetic structure of plant populations^15,16^, although in some cases long distance dispersal may negatively affect plant fitness (e.g. when seeds are dispersed outside the plant’s habitat). The probability of dispersal is also affected by diaspore size (hereafter seed size, although sometimes one or several seeds are protected by additional tissues to form the dispersal unit). Large seeds are usually associated with higher rates of establishment in low irradiation environments, as in the understory of dense forests^17^, as larger seeds may respond better than small seeds to herbivore attack^18^ and may store reserves for the critical initial stages of seedling recruitment. However, when plants evolve large seeds, the set of effective fruit dispersers tends to decrease^19^.

Hunting has profound implications on the populations of large mammals and strong effects on ecosystem dynamics^20^, and the extinction of large dispersers may result in rapid ecological and evolutionary changes, such as the reduction of seed size^21^. Similarly, the extinction of the megafauna during the Pleistocene may have represented considerable consequences on dispersal services by reducing long distance dispersal and affecting the genetic structure of plant populations over time and reducing the connectivity of large-seeded plant populations in heterogeneous landscapes^22,23^. Pires et al.^23^ proposed that extinct large-bodied animals in South America (e.g. giant sloths) frequently dispersed large seeds and estimated that long distance dispersal decreased by at least two thirds after megafaunal extinctions.

Here we show evidence that there are living frugivores that can disperse big seeds at large distances and that their dispersal roles have been understated. Hence, the first aim in this study was to summarize seed dispersal roles of oilbirds in Colombia, making emphasis on their contribution as dispersers of large seeds and describe for the first time their seed dispersal kernels. Oilbirds (*Steatornis caripensis*) are neotropical nocturnal frugivorous birds, who feed on a variety of species (mainly from the Lauraceae and Arecaceae families), and specialized on fruits with rich lipid content^24–27^. Oilbirds are known to travel long distances during the night in search for fruits^28,29^ and usually inhabit caves during the day. Their body weight varies between 400 and 450 g and the species distribution expands to central and northern South America and southern Central America.

To assess seed dispersal patterns by oilbirds, we monitored seed rain in the main cave at Cueva de Los Guacharos National Park for several years and estimated seed dispersal distances, by combining the establishment of forest plots (to identify potential parent plants) and located GPS devices on three oilbirds (to know movement patterns and estimate seed dispersal distance). The second aim was to put the maximum seed and dispersal distance in comparative context with other animals, and test the hypothesis that living animals cannot play the seed dispersal roles performed by extinct megafauna^23^.

## Results

**Figure 1:**
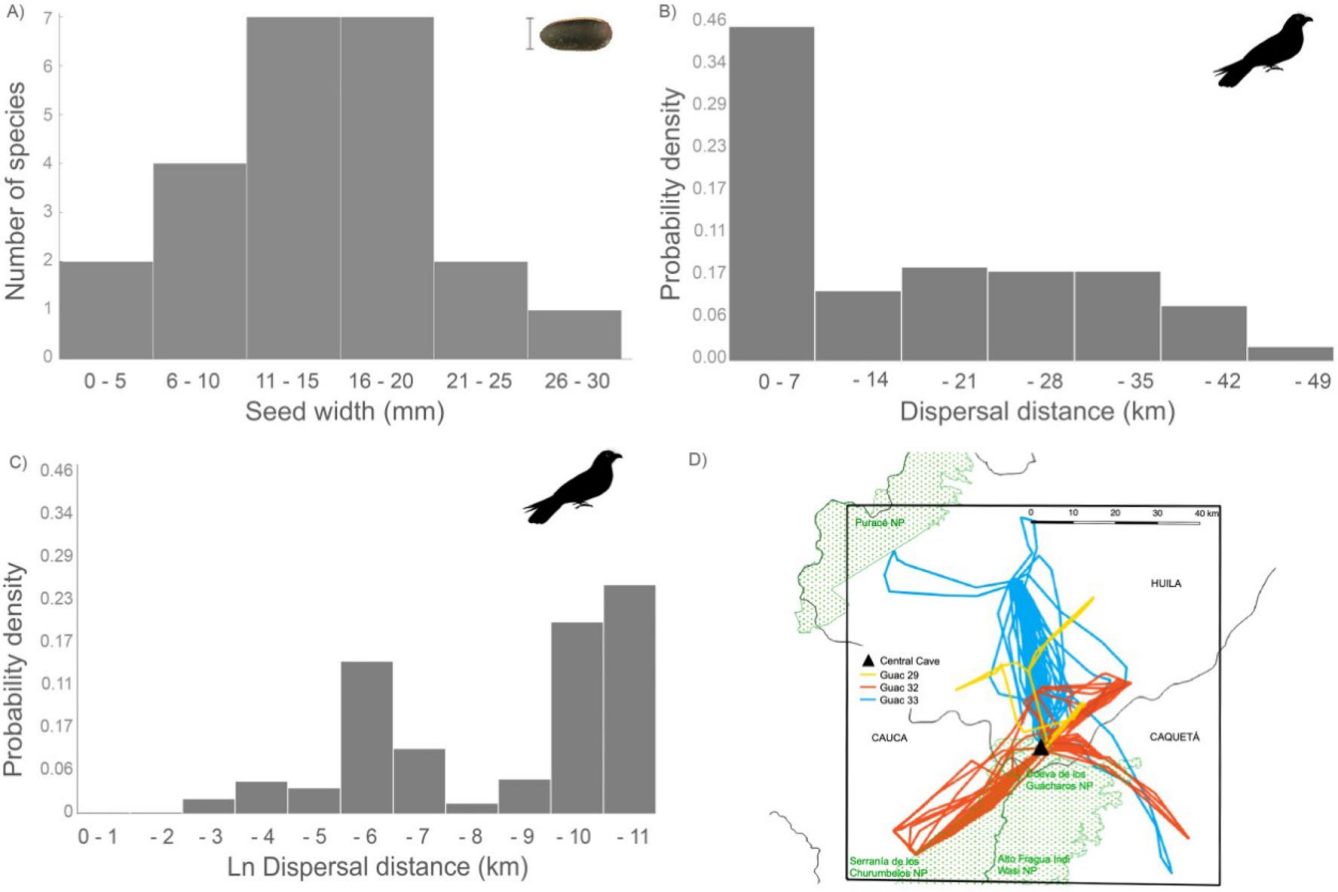
The role of oilbirds as seed dispersers. (**A**) Number of species dispersed by oilbirds in relation with the seed width. (**B**) Dispersal distances generated by oilbirds in Cueva de Los Guácharos National Park, Colombia, derived from the times of highest chance of gut delivery. (**C**) Dispersal distance with a logarithmic transformation. (**D**) Map showing the travel paths of three individuals (each with different colors, yellow corresponding to the one monitored in non-reproductive period).

Oilbirds at Cueva de Los Guacharos disperse seeds of at least 52 species, from a range of sizes. Most dispersed species have a seed width of 10–20 mm (Fig. 1A). The smallest seeds were 4 × 2.5 mm (*Hedyosmum cuatrecazanum*, Chloranthaceae) and the largest 54 × 29 mm (*Rhodostemonodaphne praeclara*, Lauraceae).

The overall distribution of dispersal distance (total kernel) by oilbirds showed a right-skewed distribution, with a peak at 0–7 km and other peaks at larger distance (e.g. 21–28 km). The distribution showed a mean dispersal distance of 10.1 km (n = 654), median of 12.8 km, mode of 15.0 km, and a range from 0 to 47.6 km (Fig. S1). The kernel derived from the times of highest probability of delivery also showed a right-skewed distribution with a mean dispersal distance of 13.6 km (n = 350) and a range from 0 to 47.6 km. (Fig. 1B). Despite the general kernel shape, only 6% and 3% of the records included seed dispersal distances less than 20 m from the parental plants, for the total and peak kernels (Fig. 1C), respectively. The kernel for regurgitated seeds also showed a similar pattern; however, in this case there was a reduction in mean dispersal distance (6.1 km, n = 301), but the range remained similar (0.001–47.6 km). Seed dispersal distance varied between the three oilbirds monitored (F = 77.3, n = 654, *p* < 0.001; Fig. 1D, Fig. S2) and the largest seeds evaluated: *Oenocarpus bataua* –Arecaceae- 35 × 18 mm, and *Dacryodes olivifera*–Burseraceae–33 × 21 mm, were dispersed up to 43.2 and 32.3 km, respectively.

**Figure. 2:**
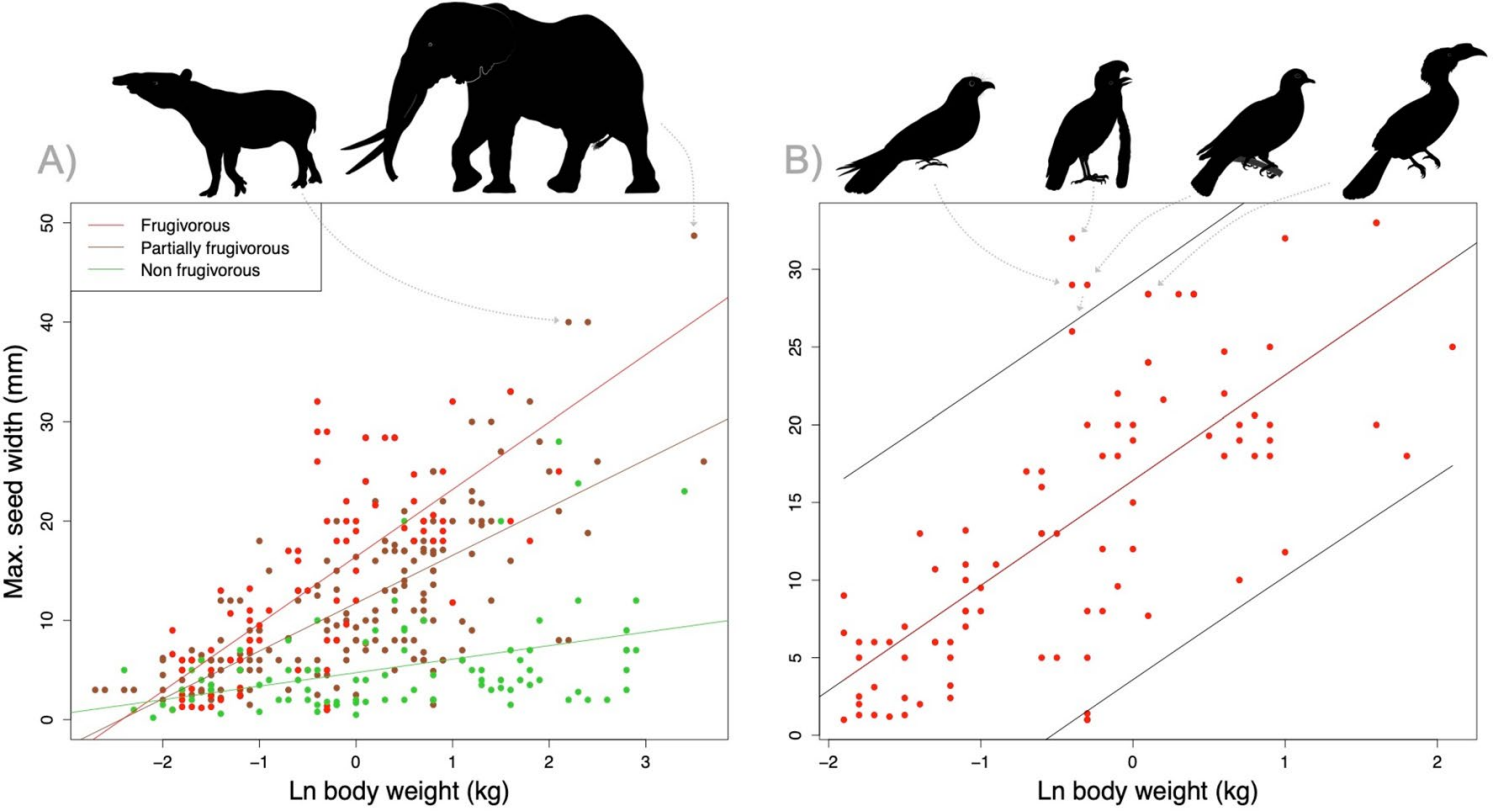
Relationship between maximum width of endozoochorous dispersed seeds and the body weight of the disperser. (**A**) Whole dataset indicating the regression lines for frugivores, partial frugivores, and non-frugivores, indicating the dots for forest elephants and tapirs. (**B**) Only frugivores, indicating oilbirds, umbrellabirds, imperial pigeons, and a hornbill. Black lines indicate prediction intervals.

We found that heavier species disperse bigger seeds (F = 91.8, n = 357, *p* < 0.001, R^2^ = 0.20). Small seed dispersers evidently disperse small seeds by endozoochory, while large dispersers may disperse large and small seeds, resulting in a triangular distribution (Fig. 2A). The predictive power of the regression was similar for frugivores and partial frugivores (both R^2^ = 0.52), and higher than for non-frugivores (R^2^ = 0.13); and it was positive in all cases (frugivores: F = 98.4, n = 94, *p* < 0.001; partial frugivores: F = 184.5, n = 170, *p* < 0.001; non-frugivores: F = 14.0, n = 93, *p* < 0.001).

Overall, elephants and tapirs were the species swallowing the largest seeds (40–50 mm in width). In fact, within the list of species showing the largest positive residuals, these large mammals were closely followed by three specialized avian frugivores (umbrella birds, oilbirds, and imperial pigeons), primate fruit specialist (spider monkeys and chimpanzees), hornbills, and the cassowary (Table 1). Considering only frugivores, the three-mentioned specialized avian frugivores stand out for the huge seeds dispersed in relation to body weight and represent outliers in this relationship (*p* = 0.02—0.004, Fig. 2B).

**Table 1:** List of species showing the largest positive residuals in the relationship between maximum seed width and body weight. The list includes frugivores (Frug) and partially frugivorous animals (P. Fr).

Plant species	Animal disperser	Diet	Residual	Max. seed width (mm)	Refs
*Balanites wilsoniana*	African elephant	P.Fr	27.5	50	^37^
*Mauritia flexuosa*	Lowland tapir	P.Fr	22.8	40	^39^
*Otoba gordonifolia*	Umberella bird	Frug	22.5	32	^29^
*Licania platypus*	Central American tapir	P.Fr	22.1	40	^40^
*Rhodostemonodaphne praeclara*	Oilbird	Frug	19.6	29	^26^
*Myristica castaneifolia*	Torresian imperial pigeon	Frug	19.3	29	^34^
*Glycydendron amazonicum*	Spider monkey	Frug	18.5	32	^38^
*Myristica iners*	Bushy-crested hornbill	Frug	17.6	28	^41^
*Beilschmiedia manii*	Chimpanzee	Frug	17.5	33	^36^
*Endiandra microneura*	Southern cassowary	Frug	17.5	33	^35^
*Myristica iners*	White crowned hornbill	Frug	17.4	28	^41^

**Figure. 3:**
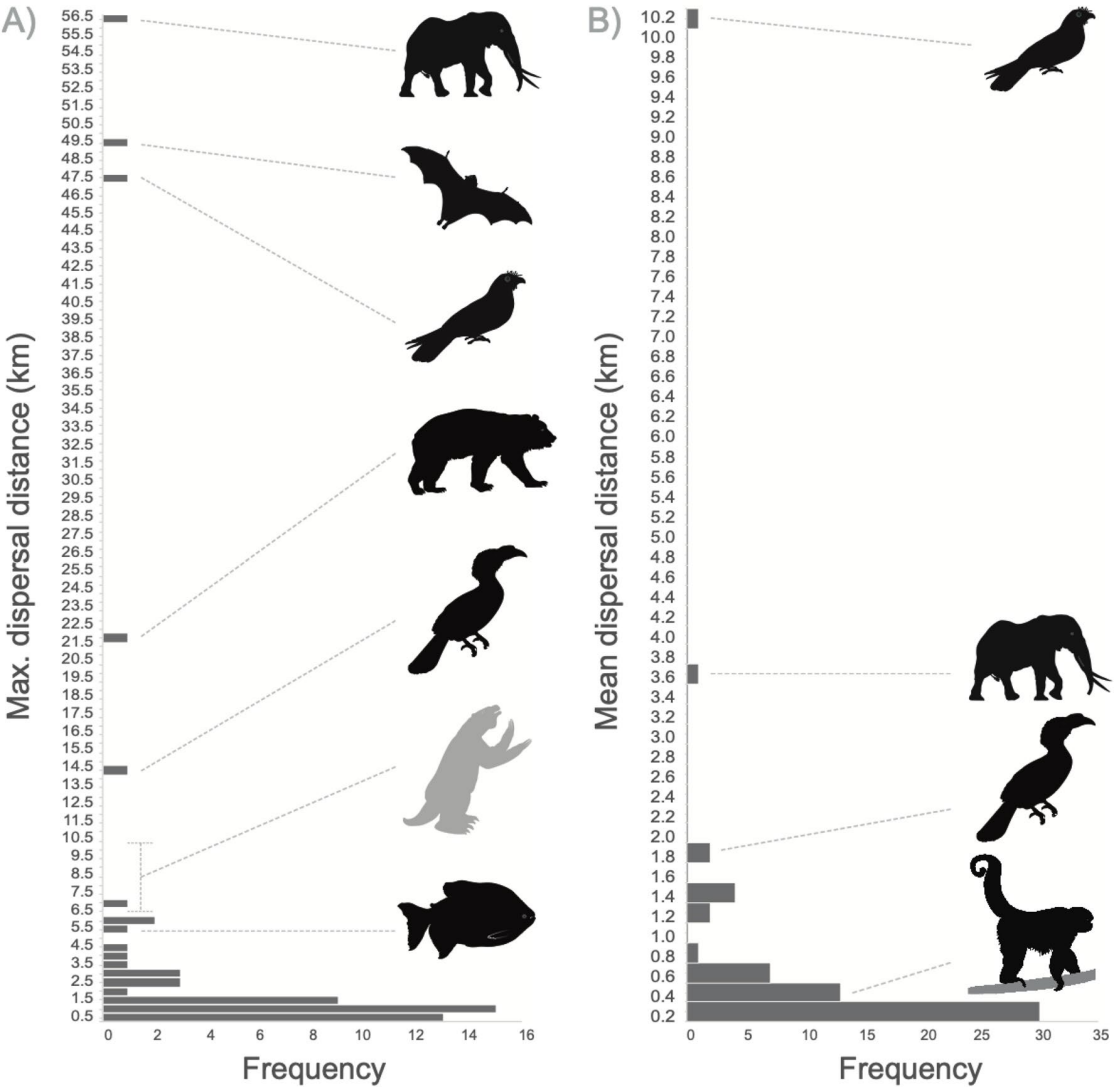
Distribution of maximum (**A**) and mean seed dispersal distances (**B**) generated by a variety of animals worldwide. The parameters corresponding to oilbirds are indicated, as extreme outliers in both cases. The estimates of extinct megafauna are also indicated by the gray giant sloth (from Figs. a7-a9 in^23^).

Maximal dispersal distance (MaxDD) (n = 62) and mean dispersal distance (MeanDD) (n = 60) demonstrated large variability between species (mean MaxDD: 4.0 km, range 0.002–57 km; mean MeanDD: 0.52 km, range 0.001–10.1 km; Fig. 3, Table S1). The maximal dispersal distance of oilbirds was the third longest estimate, after elephants (57 km) and similar to that estimated by straw-colored fruit bats (both close to 50 km) (Fig. 3A). Other extreme examples of long dispersal distance include elephants, bears, hornbills, and fish (*Colossoma macropomum*), all including estimates surpassing 5 km (Fig. 3A). The estimate for oilbirds for mean dispersal distance was far the most extreme (Fig. 3B).

## Discussion

McKey^30^ proposed that the ingestion of large seeds could have evolved as a coevolutionary process with specialized plants, that in turn evolved lipid rich pulp, allowing a high-energy fruit in the smallest possible package. On the other hand, the fact that many seeds were known to be deposited in caves, where recruitment possibilities are nil, possibly led to few studies on the roles of oilbirds as seed dispersers. However, a breakthrough occurred when Holland and collaborators^28^ found, with the use of GPS tracking devices, that oilbirds do not return every day to caves, and may spend several days foraging and dispersing seeds outside from their resting caves. Actually, the use of GPS devices allowed us to track oilbirds through the landscape and start to infer their role as seed dispersers^31,32^. Major findings include that the fruits they consume belong to forest plants, and when leaving the caves oilbirds travel on average 55 km (range 0–112 km, Fig. 1C), preferring forested areas (though they can fly over deforested lands), and avoiding the highest altitudes^31^. In addition, they disperse seeds of lowland and mountain species, and for a set of nine species, it was found that 48% of the seeds were estimated to reach suitable areas for establishment, and these estimates were higher during the non-nesting season^32^. Therefore, oilbirds may positively affect plant fitness from both quantitative and qualitative components.

In this study, we have stressed other important aspects of the quality of seed dispersal effectiveness, concluding that oilbirds are outliers in terms of the size of dispersed seeds (given their body weight) and the generated seed dispersal kernels. There was information documenting the swallowing of large seeds by oilbirds^26^, but their unusual role taking into account their small body weight was not evident before. Moreover, this is the first time that seed dispersal distances generated by oilbirds are reported, becoming the longest mean dispersal distance and the third longest maximum dispersal distance, within a data set including dozens of seed dispersers. We believe that some new discoveries will emerge as other frugivores are studied, because the advance of GPS tracking keeps providing information on unknown patterns in recent years^33^. Our study also showed that oilbirds are not the only species carrying extreme roles at swallowing large seeds and performing long distance dispersal, and emphasize the relevance of elephants, tapirs, and other specialized frugivores for their capacity to swallow large seeds^26,29,34–41^.

Seed dispersal estimates are usually based on the interplay of gut retention times and the movement patterns of the dispersers, which can be estimated using complex^42^ or simple methods^32^. As models, they involve some degree of uncertainty; however, we feel confident with the estimates shown here given that kernels generated from the full model and the model of peak delivery showed similar patterns; and even the model for regurgitated seeds showed a similar range of dispersed seeds (though with an expected reduction in mean distance). We also found differences in dispersal distance between individuals, which are partly related to the findings that oilbirds in non-reproductive periods travel less, but spend more time outside caves. Therefore, in spite of shorter dispersal distance in non-reproductive periods, plant fitness seems to increase in these periods, when oilbirds do not deposit as many seeds in caves^32^. Also, the long routes responsible for the seed dispersal patterns were consistent with the findings of oilbirds with GPS devices in Venezuela^28^, suggesting that similar dispersal services are performed by different populations of oilbirds.

We found positive associations between maximum seed size and the weight of the disperser, in contrast to a recent study that found a negative correlation^43^. This discrepancy arises mainly from the fact that we are looking just at the largest seeds (not the dimensions of all seeds reported in the diet of animal consumers) and we discarded all information concerning seeds that are predated and those not swallowed into the animal’s gut. Therefore, when removing information of small rodents or bats that are able to carry relatively large seeds in the mouth^16^ and data from seed predators that commonly destroy all the large seeds^44^, the expected positive relation emerges^45^. Our review also showed that many animal species play endozoochorous seed dispersal roles, and that large animals and super-swallowers are important and perhaps, irreplaceable providers of seed dispersal services. In addition, it showed that seed predators and specialized herbivores play some dispersal roles^46^, that should be considered when comprehensively evaluating ecosystem functioning. Similarly, this study did not take into account important synzoochorous dispersal services that also affect ecosystem dynamics^47^ and should be included in future comparisons. However, looking at the lists of animals performing the most extreme dispersal services (Table 1, Table S2), it is evident that specialized frugivores performed dominant roles. Although many other aspects of seed dispersal effectiveness should be considered in the future (as more standardized studies are done), the current information suggests that specialized frugivores play a unique role and the conservation of their populations is relevant.

We conclude that extant animals still play roles in long-distance seed dispersal, and reject the hypothesis that extinct animals were able to disperse large seeds at longer distances than extant frugivores. For instance, the maximum seed dispersal distances estimated for extinct megafauna^23^ are comparable to the mean dispersal distances generated by oilbirds when considering only regurgitated seeds by oilbirds (Fig. 3). This adds to the growing body of evidence showing that well studied megafaunal fruits are effectively dispersed by living animals^47–49^, and extinct megafauna did not necessarily disperse large seeds. In fact, in our review we detected several large herbivores that dispersed only small seeds (Table S2), which perform similar ecological roles as some of the extinct mega-herbivores and even include species in the same genera (i.e. *Equus* spp., Table S2). If the aim is to predict changes in ecosystem services related to seed dispersal, we suggest that future efforts should be focused on the study of standardized aspects of seed dispersal effectiveness for extant frugivores; and set aside the unproven idea of fruit anachronisms.

## Methods

### Field study

This study took place in Cueva de Los Guácharos National Park (1°36.14′ N; 76°8.13′ W) and surrounding areas in southern Colombia. The park has an altitudinal gradient between 1,700 and 3,000 m.a.s.l.), including primary and secondary forests^50^. The park has several caves commonly used by oilbirds, but we focused on the main cave^26^. There are at least 74 plant families of woody plants in the park^50^, and the home range of three oilbirds with GPS devices was estimated to be at least 4517 km^2^ (minimum convex polygon), then this colony has access to plants in a large area surrounding the cave^31^. We monitored seeds dispersed by oilbirds using six nylon traps (each ca. 0.64 m^2^), that were placed near the walls of the main cave in Cueva de Los Gúacharos NP^26^. We collected seeds from the trap contents for five days each month between January and December 2015, and at least once every year between 2012 and 2019. We collected 22,208 seeds from 52 plant species or morpho-species. For the most common species (N = 23), we collected and measured the width of 5–10 seeds and the mean was used to describe the seed-size distribution for the population of oilbirds (although the maximum corresponded to a single seed).

### Seed dispersal distances

To estimate seed dispersal kernels, we used seed retention times in the gut of oilbirds^51^ and movement patterns (from GPS tracking devices attached to three birds) (see details in^31^). Devices were set to provide instantaneous locations every 30 min. From this information, we constructed 25 vegetation plots in areas of heavy oilbird use within the landscape. Each plot was 0.1 ha and included all trees and palms (DBH > 10 cm). Trees of nine focal species found in the plots (trees belonging to the most abundant seed species found in the cave)^32^ were used as the starting points to set the dispersal kernels (N = 57 individuals from 9 plant species). As well, travel routes of oilbirds were used to estimate sites of seed deposition.

As seed retention in the gut is long (0.5 to 15 h^51^), we estimated three different kernels. 1. Total kernel; assumed as potential sites of deposition during 28 GPS fixes every 30 min along the whole range of gut delivery times. 2. Kernel at peak delivery times; consisting of 14 instantaneous points at the moment of higher chance of seed delivery (every 30 min between 4 and 10.5 h after fruit consumption). 3. Kernel for regurgitated seeds; assumed to be delivered between 0.5 and 3.5 h after ingestion as defecated seeds tend to spend more time in the gut than regurgitated seeds.

In cases when the GPS location was not recorded it was impossible to estimate distance; otherwise, we considered an estimate for each event, independent of the number of seeds that could reach a site (to avoid pseudoreplication). Each dispersal distance was estimated as the horizontal distance between the feeding tree and the GPS fix, using the package “adehabitat”^52^ in R software^53^.

### Comparison with other animals

We based our comparison on recent articles comparing seed size with the body weight of consumers and others comparing dispersal kernels for different frugivores^33,54^. Furthermore, we searched in Google Scholar and Web of Knowledge between March and May 2020, using the following keywords: seed dispersal and seed size, seed dispersal and seed width, seed dispersal and seed diameter, seed dispersal distance and frugivor*. We reviewed approximately 3489 articles, choosing only those reporting endozoochoric seed dispersal and the size of seeds or the list of species being dispersed. We discarded articles documenting frugivory in one or few tree species, studies focusing on strict seed predators, studies with limited sample size, and studies not differentiating between endozoochory and synzoochory (maintaining ca. 500 studies). We based our review on studies focusing on one or few seed dispersers, preferentially including analyses of fecal samples or those explicitly mentioning seed handling technics. We assembled a database including the species of the animal consumer, its body weight (as an average between sexes), the width or diameter of the largest seed swallowed, and the plant species it belongs. When these data were not in the original articles, the seed width was estimated from images available in several Herbaria and plant databases (e.g. Tropicos.org, Neotropical Plant Specimens, Global Plants-JStor) or the information was received directly from authors. To avoid pseudoreplication, only one record was kept for each animal species (final n = 357 species), corresponding to the studies reporting the largest seed. We used a broad dietary classification for each animal (frugivorous: when overall > 60% of the diet corresponds to fruits, partially frugivorous: 60–10%, and non-frugivorous: < 10%). From the studies reporting dispersal kernels, we gathered information on mean and maximum dispersal distance. When several studies were available for one animal species, we kept the study showing the longest estimates.

### Data analyses

We constructed frequency distributions for the width of seeds dispersed by oilbirds, dispersal kernels and both mean and maximum dispersal distance found in the literature. We used ANOVA to compare dispersal distances generated by the three oilbirds. Regression analyses were used to explain the maximum seed width (in mm) from the body weight of dispersers (in kg), which was log-transformed, for the full dataset and each dietary classification). We detected outliers in this relationship using the outlier test function from “car” package R^55^, and we listed the species with the highest and the lowest abilities to swallow large seeds given their body weight from the residuals of the relationship.

## Supplementary Information

Supplementary Information 1.

Supplementary Information 2.
