# Supplementary material for: Oilbirds disperse large seeds at longer distance than extinct megafauna
Source: Sci Rep. 2021 Jan 11;11:420. doi: 10.1038/s41598-020-79280-4 (PMC7801487; doi:10.1038/s41598-020-79280-4)
Supplement: Supplementary file 1 — Supplementary Information 1. [file 41598_2020_79280_MOESM1_ESM.pdf]

Supplementary Materials**: Oilbirds disperse large seeds at longer distance than extinct megafauna**

Authors: Pablo R. Stevenson, Laura Cardona, Sasha Cárdenas, Andrés Link.

Fig. S1.

Full model of dispersal distances generated by oilbirds in Cueva de Los Guácharos National Park, Colombia.

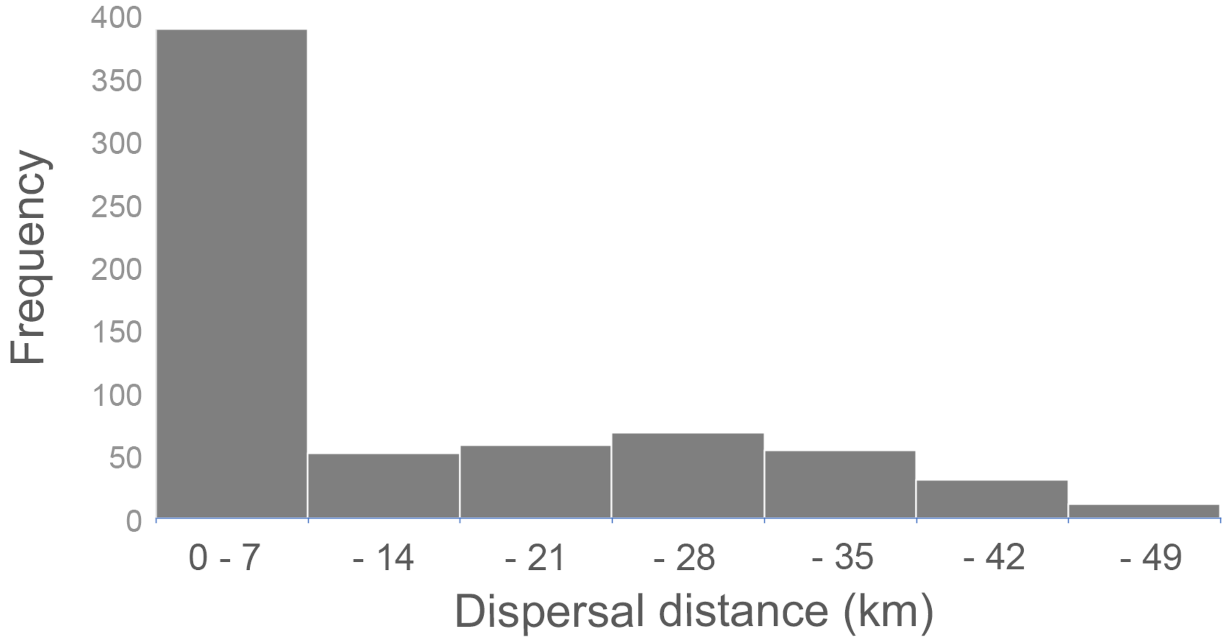

Fig. S2.

Comparison of seed dispersal distance generated by three oilbirds at Cueva de Los Guácharos National Park (Colombia). Individual 29 operated outside the nesting period, while the other two were active during the nesting period.

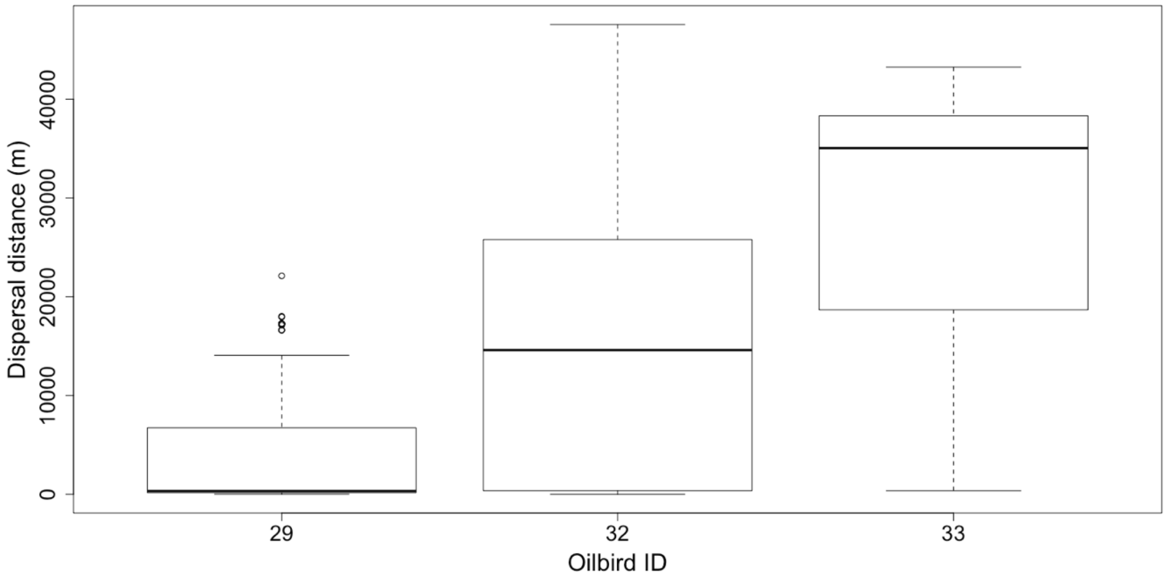

Table S1.
List of species generating seed dispersal at extreme long distances.

Scientific name	Common name	Max. dispersal distance (m)	Mean DD (m)	Reference
Loxodonta africana	African Elephant	57000	3500	(1)
Eidolon helvum	Straw-coloured fruit bat	49700	54	(2)
Steatornis carpipensis	Oilbird	47584	10085	This study
Ursus thibetanus	Japanese black bear	22000	1250	(3)
Bycanistes bucinator	Trumpeter hornbills	14500	512	(4)
Ceratogymna atrata	Black-casqued hornbill	6919	1685	(5)
Elephas maximus	Asian elephant	5772	1664	(6)
Ceratogymna cylindricus	White-thighed hornbill	5698	1374	(5)
Colossoma macropomum	Tambaqui	5495	445	(7)
Chelonoidis porteri	Galapagos tortoise	4355	394	(8)

Table S2.
List of species showing the highest negative residuals in the relationship between maximal seed width and the body weight of the consumer. None of the listed animals are frugivores.

Scientific name	Common name	Residual	Reference
Capreolus capreolus	Roe deer	-11.5	(9)
Orycteropus afer	Aardvark	-11.6	(10)
Bos taurus	Galloway cattle	-11.8	(11)
Capra hircus	Goat	-11.9	(12)
Equus caballus	Horse	-12.0	(11)
Axis porcinus	Indian hog deer	-12.2	(13)
Bos gaurus	Gaur	-12.3	(14)
Ovis aries	Domestic sheep	-12.4	(15)
Dama dama	Fallow deer	-12.4	(9)
Antidorcas marsupialis	Springbok	-12.5	(10)
Bison bonasus	European bison	-13.9	(16)
Equus africanus	Donkey	-14.0	(17)
Connochaetes taurinus	Wildebeest	-14.7	(18)
Ovibos moschatus	Muskox	-15.3	(19)
Bison bison	American bison	-15.9	(20)
Equus burchelli	Zebra	-15.9	(18)

References

1. Blake, S., Deem, S. L., Mossimbo, E., Maisels, F. & Walsh, P. Forest elephants: Tree planters of the Congo. *Biotropica* **41**, 459-468 (2009).
2. Abedi-Lartey, M., Dechmann, D. K. N., Wikelski, M., Scharf, A. K. & Fahr, J. Long-distance seed dispersal by straw-coloured fruit bats varies by season and landscape. *Glob. Ecol. Conserv.* **7**, 12-24 (2016).
3. Koike, S. & Masaki, T. Characteristics of fruits consumed by mammalian frugivores in Japanese temperate forest. *Ecol. Res.* **34**, 246-254 (2019).
4. Lenz, J. *et al.* Seed-dispersal distributions by trumpeter hornbills in fragmented landscapes. *Proceedings of the Royal Society B-Biological Sciences* **278**, 2257-2264 (2011).
5. Whitney, K. D. *et al.* Seed dispersal by *Ceratogymna* hornbills in the Dja Reserve, Cameroon. *J. Trop. Ecol.* **14**, 351-371 (1998).
6. Kitamura, S., Yumoto, T., Poonswad, P. & Wohandee, P. Frugivory and seed dispersal by Asian elephants, *Elephas maximus*, in a moist evergreen forest of Thailand. *J. Trop. Ecol.* **23**, 373-376 (2007).

7. Anderson, J. T., Nuttle, T., Saldaña Rojas, J. S., Pendergast, T. H. & Flecker, A. S. Extremely long-distance seed dispersal by an overfished Amazonian frugivore. *Proc. R. Soc. Lond., Ser. B: Biol. Sci.* **278**, 3329 - 3335 (2011).
8. Blake, S. *et al.* Seed dispersal by Galapagos tortoises. *J. Biogeogr.* **39**, 1961-1972 (2012).
9. Panter, C. J. & Dolman, P. M. Mammalian herbivores as potential seed dispersal vectors in ancient woodland fragments. *Wildl. Biol.* **18**, 292-303 (2012).
10. Milton, S. J. & Dean, W. R. J. Seeds dispersed in dung of insectivores and herbivores in semi-arid southern Africa. *J. Arid Environ.* **47**, 465-483 (2001).
11. Cosyns, E., Claerbout, S., Lamoot, I. & Hoffmann, M. Endozoochorous seed dispersal by cattle and horse in a spatially heterogenous landscape. *Plant Ecol.* **178**, 149-162 (2005).
12. Heleno, R., Blake, S., Jaramillo, P., Traveset, A., Vargas, P., & Nogales, M. Frugivory and seed dispersal in the Galapagos: what is the state of the art? *Integrative Zoology* **6**, 110-129 (2011).
13. Davis, N. E., Forsyth, D. M., & Coulson, G. Facilitative interactions between an exotic mammal and native and exotic plants: hog deer (Axis porcinus) as seed dispersers in south-eastern Australia. *Biol. Invasions* **12**, 1079-1092 (2010).
14. Sridhara, S., McConkey, K., Prasad, S., & Corlett, R. T. Frugivory and Seed Dispersal by Large Herbivores of Asia i*n The ecology of large herbivores in souht and southeast Asia.* (eds. Ahrestani, F. S., Sankaran, M.) 121-150. (Dordrecht: Springer, 2016).
15. Horn, A., Pachmann, G. & Poschlod, P. Can sheep replace indigenous antelope as seed dispersers in the Kalahari? *J. Arid Environ.* **91**, 69-78 (2013).
16. Jaroszewicz, B., Pirozikow, E. & Sagehorn, R. Endozoochory by European bison (Bison bonasus) in Bialowieza Primeval Forest across a management gradient. *For. Ecol. Manage.* **258**, 11-17 (2009).
17. Couvreur, M., Cosyns, E., Hermy, M. & Hoffmann, M. Complementarity of epi- and endozoochory of plant seeds by free ranging donkeys. *Ecography* **28**, 37-48 (2005).
18. Shiponeni, N. N. & Milton, S. J. Seed dispersal in the dung of large herbivores: implications for restoration of Renosterveld shrubland old fields. *Biodivers. Conserv.* **15**, 3161-3175 (2006).
19. Bruun, H. H., Lundgren, R. & Philipp, M. Enhancement of local species richness in tundra by seed dispersal through guts of muskox and barnacle goose. *Oecologia* **155**, 101-110 (2008).
20. Rosas, C. A., Engle, D. M., Shaw, J. H. & Palmer, M. W. Seed dispersal by *Bison bison* in a tallgrass prairie. *Journal of Vegetation Science* **19**, 769-778 (2008).
