# Supplementary material for: Oilbirds disperse large seeds at longer distance than extinct megafauna
Source: Sci Rep. 2021 Jan 11;11:420. doi: 10.1038/s41598-020-79280-4 (PMC7801487; doi:10.1038/s41598-020-79280-4)
Supplement: Supplementary file 2 — Supplementary Information 2. [file 41598_2020_79280_MOESM2_ESM.pdf]

Oilbirds disperse large seeds at longer distance than extinct megafauna

Pablo R. Stevenson, Laura Cardona, Sasha Cárdenas and Andrés Link.

Data S1. Database used to compare the maximum size of seeds swallowed by a variety of animals (maximum seed width in mm); as well as maximum and mean seed dispersal distances (m). The database includes: Species name, common name, dietary classification, body weight (kg), seed width (mm), plant species, reference on seed dispersal, maximum dispersal distance (Max. DD), mean dispersal distance (Mean DD), and the reference for seed dispersal kernels (if different from the first citation).

Notes: Only one record was used for each species, the one showing the most extreme value of seed size or dispersal distance. If you are aware of other species that could be included, or more extreme values of seed width or dispersal distance for the listed species, please contact us (pstevens@uniandes.edu.co) and let us now the information. An exel version (without complete refrerences) will be available at https://www.asoprimatologicacolombiana.org.

Sepecies	Common name	Diet	Body weight (kg)	Max. seed width (mm)	Plant species	Reference	Max. dispersal distance (m)	Mean dispersal distance (m)	Distance Reference
Aceros comatus	White crowned hornbill	Fru.	1.4	28.4	*Myristica iners*	Kitamura et al. 2011			
Aceros corrugatus	Wrinkled hornbill	Fru.	1.6	21.6	*Horsfieldia sucosa*	Kitamura et al. 2011			
Aceros undulatus	Wreathed hornbill	Fru.	2.4	28.4	*Myristica iners*	Kitamura et al. 2011			
Aepyceros melampus	Impala	N.F.	48	6	*Acacia nilotica*	Slater & du Toit 2002			
Akodon paranaensis	Small rodent	N.F.	0.03	3	*Cyphomandra sp.*	Casella & Cáceres 2006			
Aldabrachelys gigantea	Giant tortoise	P.F.	250	18.8	*Veitchia merrillii*	Waibel et al. 2013			
Alopecoenas xanthonurus	Mariana fruit dove	Fru.	0.09	8	*Discocalyx megacarpa*	Fricke et al. 2019			
Alophoixus pallidus	Puff-throated bulbul	Fru.	0.04	10.7	*Phoebe cathia*	Kitamura et al. 2002	454	28	Khamcha et al. 2014
Alouatta caraya	Golden-mantled howler	P.F.	7	15	*Brosimum cf. alicastrum*	Moura & McConkey 2007	360	40	
Alouatta guariba	Brown howler	P.F.	5.2	12	*Neomithrantes obscura*	Martins 2008			
Alouatta palliata	Howler monkey	P.F.	7	18	*Spondias mombin*	Wehncke et al. 2004	811	112	
Alouatta pigra	Howler monkey	P.F.	7	17	*Virola guatemalensis*	Benitez-Malvido et al. 2014	439	149	Zarate et al. 2014
Alouatta seniculus	Red howler	P.F.	6.4	25	*Pouteria laevigata*	Julliot 1996	637	239	
Anas acuta	Northern pintails	N.F.	0.93	4	*Sparganium eurycarpum*	Mueller & van der Valk 2002			
Anas castanea	Chestnut teal	N.F.	0.64	1.8	*Triglochin striata*	Raulings et al. 2011			
Anas crecca	Eurasian teal	N.F.	0.35	3	*Echinochloa crus-galli*	Brochet et al. 2009			
Anas gracilis	Grey teal	N.F.	0.51	1.8	*Bolboschoenus fluviatilis*	Green et al. 2008	1000		Raulings et al. 2011
Anas platyrhynchos	Mallard duck	N.F.	1.18	7.8	*Iris pseudacorus*	Kleyheeg & van Leeuwen 2015			
Anas superciliosa	Pacific black duck	N.F.	1.22	1.8	*Triglochin striata*	Raulings et al. 2011	750		
Anodorhynchus leari	Lear's Macaw	N.F.	0.94	1.73	*Cereus jamacaru*	Blanco et al. 1916			
Anolis garmani	Anolis lizard	P.F.	0.002	3	*Canella winterana*	Herrel et al. 2004			

Sepecies	Common name	Diet	Body	Seed	Plant species	Reference	Max. DD	Mean DD	Distance Reference
Anolis grahami	Anolis lizard	P.F.	0.004	3	*Canella winterana*	Herrel et al. 2004			
Anolis lineatopus	Anolis lizard	P.F.	0.003	3	*Canella winterana*	Herrel et al. 2004			
Anolis valencienni	Anolis lizard	P.F.	0.005	3	*Canella winterana*	Herrel et al. 2004			
Anorrhinus galeritus	Bushy-crested hornbill	Fru.	1.2	28.4	*Myristica iners*	Kitamura et al. 2011			
Anthornis melanura	Bellbird	P.F.	0.03	4.5	*Ilex aquifolium*	Williams & Karl 1996			
Anthracoceros albirostris	Oriental pied hornbill	Fru.	0.74	18	*Horsfieldia kingii*	Datta & Rawat 2003			
Antidorcas marsupialis	Springbok	N.F.	39	1.5	*Asteraceae*	Milton & Dean 2001			
Apalopteron familiare	Bonin Islands white-eye	P.F.	0.02	5		Kawakami et al. 2009			
Aplonis opaca	Micronesian starling	P.F.	0.08	9	*Aglaia mariannensis*	Fricke et al. 2019			
Arctictis binturong	Binturong	P.F.	14.5	16.7	*Platymitra macrocarpa*	Kitamura et al. 2002			
Arion rufus	Red slug	N.F.	0.02	2	*Anemone nemorosa*	Türke & Weisser 2013			
Arremon flavirrostris	Saffron-billed sparrow	N.F.	0.03	3.5	*Morus nigra*	Gianini 1999			
Artibeus jamaicensis	Jamaica fruit-eating bat	Fru.	0.04	2	*Maclura tinctoria*	Heithaus et al. 1975			
Artibeus lituratus	Great fruit-eating bat	Fru.	0.07	3.2	*Psidium guajava*	Zapata-Mesa et al. 2017			
Artibeus planirostris	Flatfaced fruiteating bat	Fru.	0.05	6	*Celtis iguanaeus*	Sanchez et al. 2012			
Asio flameus	Short-eared owl	N.F.	0.4	0.8	*Chamaesyse sp.*	Heleno et al. 2011			
Ateles belzebuth	Spider monkey	Fru.	8	25	*Iryanthera sp.*	Dew 2008	1281	453	Link & DiFiore 2006
Ateles chamek	Black spider monkey	Fru.	8	18	*Socratea exorrhiza*	Palma & Stevenson 2010	1500	245	Russo et al. 2006
Ateles geoffroyi	Spider monkey	Fru.	7	18	*Spondias mombin*	Benitez-Malvido et al. 2014	345		
Ateles hybridus	Brown spider monkeys	Fru.	7.6	19	*Pouteria sp.*	Link et al. 2012			
Ateles paniscus	Spider monkey	Fru.	9.5	32	*Glycydendron amazonicum*	Guillotin et al. 1994			
Atlantoxerus getulus	Barbary ground squirrel	N.F.	0.33	2	*Lycium intricatum*	Lopez-Dárias & Nogales, 2008			
Atlapetes citrinellus	Yellow-striped brush-finch	Fru.	0.028	6	*Alophyllus edulis*	Ruggera et al. 2014			
Auchenipterichthys longimanus	Amazonian catfish	P.F.	0.112	18	*Licania sp.*	Mannheimer et al. 2003			

Sepecies	Common name	Diet	Body	Seed	Plant species	Reference	Max. DD	Mean DD	Distance Reference
Axis porcinus	Indian hog deer	N.F.	37	3	*Acacia longifolia*	Davis et al. 2005			
Baillonius bailloni	Saffron Toucanet	P.F.	0.14	15	*Maytenus robusta*	Galleti et al. 2000			
Bison bison	American bison	N.F.	587.5	3	*Cephalanthus occidentalis*	Rosas et al. 2008			
Bison bonasus	European bison	N.F.	610	5	*Malus domestica*	Jaroszewicz et al. 2009			
Bombycilla cedrorum	Cedar waxwing	Fru.	0.032	7	*Prunus serotina*	Witmer 1996			
Bos gaurus	Gaur	N.F.	850	7		Srinhara et al. 2016			
Bos taurus	Galloway cattle	N.F.	562.5	7	*Ligustrum vulgare*	Cosyns et al. 2005			
Brachyteles arachnoides	Southern muriqui	P.F.	9.5	20	*Hymenaea courbaril*	Martins 2008	693	169	
Bradypus torquatus	Maned sloth	N.F.	5.7	2	*Cecropia hololeuca*	Chiarello 1998			
Brycon amazonicus	Yamu	P.F.	1	16.4	*Calophyllum sp.*	Correa et al. 2015			
Brycon hilarii	Piraputanga	P.F.	3.4	13.5	*Pouteria glomerata*	Correa et al. 2015			
Brycon melanopterus	Amazonic tarpon	P.F.	4.4	16	*Swartzia sp.*	Correa et al. 2015			
Buceros bicornis	Great hornbill	Fru.	2.6	28.4	*Myristica iners*	Kitamura et al. 2011			
Buceros rhinoceros	Rhinoceros hornbill	Fru.	2.2	28.4	*Myristica iners*	Kitamura et al. 2011			
Buceros vigil	Helmeted hornbill	Fru.	3	19.3	*Horsfieldia tomentosa*	Kitamura et al. 2011			
Bycanistes bucinator	Trumpeter hornbills	Fru.	0.725			Lenz et al. 2011	14500	512	
Cacajao melanocephalus	Black-headed uacari	N.F.	3	20	*Sloanea synandra*	Boubli 1999			
Callicebus coimbrai	Titi monkey	P.F.	1.2	7	*Protium heptaphyllum*	Baiao et al. 2015		139	
Callosciurus erythraeus	Pallas's squirrel	N.F.	0.27	4	*Schinus molle*	Bobadilla et al. 2016			
Caluromys philander	Bare-tailed woolly opossum	Fru.	0.265	5	*Amaioua guianensis*	Lessa & da Costa 2010			
Camarhynchus parvulus	Small tree finch	N.F.	0.013	1	*Kyllingia breviflora*	Heleno et al. 2011			
Capra hircus	Goat	N.F.	35	3.2	*Psidium guajava*	Heleno et al. 2011			
Capreolus capreolus	Roe deer	N.F.	22.5	3	*Vicia sativa*	Panter & Dolman 2012			
Carolia perspicillata	Seba's short-tailed bat	Fru.	0.015	2	*Maclura tinctoria*	Heithaus et al. 1975			
Carollia castanea	Short-tailed fruit bat	Fru.	0.013	1	*Cecropia angustifolia*	Zapata-Mesa et al. 2017			

Sepecies	Common name	Diet	Body	Seed	Plant species	Reference	Max. DD	Mean DD	Distance Reference
Carollia sowelli	Sowell's short-tailed bat	Fru.	0.015	2.5	*Senna quinquangulata*	Castro-Luna & Galindo 2012			
Casuarius casuarius	Southern cassowary	Fru.	44	33	*Entiandra microneura*	Stocker & Irvine 1983	1000	337	Wescott et al. 2005
Catharus aurantiirostris	Orange-billed Nightingale-thrush	P.F.	0.026	2.8	*Tournefortia bicolor*	Lindell et al. 2013			
Cebus capucinus	Capuchin monkey	P.F.	2.75	13	*Ximenia americana*	Wehncke et al. 2004	844	216	
Cephalophus callipygus	Peter's duiker	P.F.	16	23	*Antocaryon klaineanum*	Feer 1995			
Cephalophus dorsalis	Bay duiker	P.F.	19.5	20	*Gambeya beguei*	Feer 1995			
Cephalophus sylvicultor	Yellow backed duiker	P.F.	62.5	32	*Irvingia gabonensis*	Feer 1995			
Cephalopterus penduliger	Long-wattled umbrellabird	Fru.	0.445	32	*Otoba gordonifolia*	Karubian et al. 2012	444	2008	
Ceratogymna atrata	Black-casqued hornbill	Fru.	1.2	24	*Dacryodes edulis*	Holbrook & Smith 2000	6919	1685	Whitney et al. 1998
Ceratogymna cylindricus	White-thighed hornbill	Fru.	1.2	24	*Dacryodes edulis*	Holbrook & Smith 2000	5698	1374	Whitney et al. 1998
Ceratogymna fistulator	Piping hornbill	Fru.	0.5865	18	*Canarium schweinfurthii*	Whitney et al. 1998			
Cercopithecus ascanius	Redtail monkeys	P.F.	3.9	6.1	*Prunus africana*	Farwig et al. 2006			
Cercopithecus cephus	Moustached guenon	Fru.	3.7	18	*Vitex grandifolia*	Poulsen et al. 2001			
Cercopithecus l'hoesti	Mountain monkey	P.F.	4.8	6	*Canthium sp.*	Kaplin & Moermond 1998			
Cercopithecus lhoesti	L'Hoest's monkeys	P.F.	4.8	6	*Canthium* sp.	Kaplin & Moermond 1998			
Cercopithecus mitis	Blue monkey	P.F.	8.6	6	*Canthium sp.*	Kaplin & Moermond 1998			
Cercopithecus nictitans	White-nosed guenon	P.F.	5.3	16.9	*Santiria trimera*	Chapman et al. 2010			
Cercopithecus petaurista	White-nosed guenon	P.F.	3.3	17	*Canarium schweinfurthii*	Poulsen et al. 2001			
Cercopithecus pogonias	Crowned guenon	P.F.	3.6	18	*Vitex grandifolia*	Poulsen et al. 2001			
Cerdocyon thous	Crab-eating fox	P.F.	6.1	10	*Allagoptera arenari*	Gatti et al. 2006			
Cervus elaphus	Red deer	N.F.	70.5	5	*Tilia cordata*	Jaroszewicz et al. 2013			
Cervus nippon	Sika deer	N.F.	42	7	*Diospyros japonica*	Yamashiro & Yamashiro 2006			
Cervus unicolor	Sambar	N.F.	180	23.8	*Spondias pinnata*	Kitamura et al. 2002			

Sepecies	Common name	Diet	Body	Seed	Plant species	Reference	Max. DD	Mean DD	Distance Reference
Chamaepetes unicolor	Black guan	Fru.	0.95	20	*Beilschmiedia pendula*	Wheelwright et al. 1984			
Cheirogaleus major	Greater dwarf lemurs	P.F.	0.43	12.5	*Cryptocarya sp.*	Lahann 2007			
Cheirogaleus medius	Fat-tailed dwarf lemur	P.F.	0.19	8.2	*Erythroxylon sp.*	Lahann 2007			
Chelonoides denticulata	Amazoniana tortoise	P.F.	15	30	*Astrocaryum murumuru*	Guzman & Stevenson 2008	1100	226	Jerozolimski et. 2009
Chelonoidis chilensis	Chaco tortoise	P.F.	1.5	8	*Ziziphus mistol*	Varela & Bucher 2002a			
Chelonoidis porteri	Galapagos tortoise	N.F.	80	10	*Hippomane mancinella*	Blake et al. 2015	4355	394	Blake et al. 2012
Chiroxiphia boliviana	Yungas manakin	P.F.	0.02	6	*Tapirira guianensis*	Montaño-Centellas 2012			
Chlorocebus tantalus	Vervet monkey	P.F.	5.5	6.8	*Landolphia spp.*	Agmen et al. 2009			
Chlorospingus ophthalmicus	Common bush-tanager	P.F.	0.01	4	*Cinnamomum porphyrium*	Blendinger et al. 2015			
Chrysetnys picta	Eastem painted turtle	P.F.	0.4	2.5	*Nuphar lutea*	Padgett et al. 2010			
Chrysocyon brachyurus	Maned wolf	P.F.	23	20	*Syagrus romazoffianum*	Bueno & Motta-Junior 2009			
Cinnyricinclus leucogaster	Violet-backed starling	Fru.	0.05	6	*Prunus africana*	Farwig et al. 2006			
Civettictis civetta	African civet	P.F.	13	9.9		Penje 1994			
Cleptornis marchei	Golden white-eye	P.F.	0.02	6	*Planchonella obovata*	Fricke et al. 2019			
Colaptes pitius	Chilean flicker	P.F.	0.15	6	*Schinus polygamus*	Reid & Armesto 2011			
Colobus guereza	Black-and-white colobus	N.F.	14	6	*Prunus africana*	Farwig et al. 2006			
Colorhamphus parvirostris	Patagonian tyrant	P.F.	0.01	6	*Maytenus boaria*	Reid & Armesto 2011			
Colossoma macropomum	Tambaqui	P.F.	1.7	22	*Astrocaryum jauari*	Lucas 2008	5495	445	Anderson et al. 2011
Connochaetes taurinus	Wildebeest	N.F.	165	2	*Romulea rosea*	Shiponeni & Milton 2006			
Conolophus subcristatus	Galapagos land iguana	P.F.	6.5	1.5	*Solanum lycopersicum*	Heleno et al. 2011			
Coracopsis nigra	Lesser vasa parrot	N.F.	0.19	8	*Commiphora guillaumini*	Böhning-Gaese et al. 1999			
Corvus corax	Raven	P.F.	1.35	10	*Laurus azorica*	Nogales & Hernandez 1994			
Corvus frugilegus	Rook	P.F.	0.43	4.5	*Cornus alba*	Czarnecka & Kitowski 2010			
Corvus macrorhynchos	Large-billed crow	P.F.	0.74	7	*Magnolia obovata*	Nishi & Tsuyuzaki 2004			
Corythaeola cristata	Great blue turaco	Fru.	1	19	*Stromboscia scheffleri*	Sun et al. 1997			

Sepecies	Common name	Diet	Body	Seed	Plant species	Reference	Max. DD	Mean DD	Distance Reference
Crotophaga ani	Smooth-billed ani	N.F.	0.11	3.5	*Tournefortia rufosericea*	Heleno et al. 2011			
Ctenosaura hemilopha	Spiny-tailed iguana	N.F.	3	9	*Cyrtocarpa edulis*	Blázqeuz & Rodriguez 2007			
Ctenosaura similis	Black spiny-tailed iguana	P.F.	2	13	*Spondias purpurea*	Traveset 1990			
Cyanistes teneriffae	Canary Island blue tit	N.F.	0.01	1.5	*Rubus ulmifolius*	González-Castro et al. 2013b			
Cygnus atratus	Black swan	N.F.	6.2	2	*Polygonum arenastrum*	Green et al. 2008			
Dama dama	Fallow deer	N.F.	63	3.5	*Triticum spp.*	Panter & Dolman 2012			
Didelphis albiventris	White-eared opossum	P.F.	0.9	8	*Passiflora sp.*	Cáceres 2002			
Diplothrix legata	Ryukyu long-furred rat	N.F.	0.58	1.5	*Oreocnide pedunculata*	Nago et al. 2019			
Dromaius novaehollandiae	Emu	P.F.	34.2	27	*Macrozamia fraseri*	Calviño-Cancela et al. 2006	2500	540	Nield et al. 2020
Dromiciops gliroides	Colocolo opossum	P.F.	0.03	6	*Myoschilos oblongum*	Amico et al. 2009			
Ducula badia	Mountain imperial pigeon	Fru.	0.55	20		Kitamura et al. 2002			
Ducula badia	Mountain imperial pigeon	Fru.	0.63	12	*Mastixia pentranda*	Sankamethawee et al. 2011			
Ducula pacifica	Pacific pigeons	Fru.	0.39	26	*Myristica hypagyrarea*	McConkey 2000			
Ducula spilorrhoa	Torresian imperial pigeon	Fru.	0.5	29	*Myristica castanaefolia*	Wood 1924			
Eidolon dupreanum	Madagascan fruit bat	Fru.	0.3	5	*Polyscias sp.*	Picot et al. 2007			
Eidolon helvum	Straw-coloured fruit bat	Fru.	0.29	13	*Garcinia buchananii*	Webala et al. 2014	49700	54	Abedi-L et al. 2016
Elaenia albiceps	White-crested Elaenia	P.F.	0.02	6	*Schinus polygamus*	Reid & Armesto 2011			
Elaenia flavogaster	Yellow-bellied Elaenia	P.F.	0.02	3	*Lantana camara*	Lindell et al. 2013			
Elaenia sptrepera	Slaty elaenia	N.F.	0.02	5	*Blepharocalyx salicifoius*	Gianini 1999			
Elephas maximus	Asian elephant	P.F.	4050	26	*Spondias pinnata*	Kitamura et al. 2007	5772	1664	
Equus africanus	Donkey	N.F.	202	2.8	*Vicia cracca*	Couvreur et al. 2005			
Equus burchelli	Zebra	N.F.	400	2	*Romulea rosea*	Shiponeni & Milton 2006			

Sepecies	Common name	Diet	Body	Seed	Plant species	Reference	Max. DD	Mean DD	Distance Reference
Equus caballus	Horse	N.F.	690	7	*Ligustrum vulgare*	Cosyns et al. 2005			
Eulemur fulvus	Brown lemur	P.F.	2.5	17.6	*Astrotrichilia asterotricha*	Sato 2012	1077	124	Sato 2018
Eulemur macaco	Black lemur	P.F.	1.9	18	*Diospyros clusiifolia*	Birkinshaw 2001			
Eulemur rubriventer	Red bellied lemur	P.F.	1.85	17	*Ravensara sp.*	Dew & Wright 1998	359	120	Razafindratsima 2014
Eulemur rufrifrons	Red fronted brown lemur	P.F.	2.25	17	*Cryptocarya crassifolia*	Razafindratsima et al. 2014	417	96	
Euphonia cyanocephala	Golden-rumped Euphonia	Fru.	0.01	5	*Eugenia uniflora*	Blendinger et al. 2015			
Euphractus sexcinctus	Yellow armadillo	N.F.	4.8	10	*Hancornia speciosa*	Dalponte & Tavares-Filho 2004			
Fulica atra	Eurasian coot	N.F.	0.9	0.5	*Typha orientalis*	Green et al. 2008			
Gallirallus australis	Weka	Fru.	0.9	12	*Elaeocarpus dentatus*	Carpenter et al. 2019	2333	135	
Gallotia atlantica	Atlantic lizard	P.F.	0.04	2	*Lycium intricatum*	Lopez-Dárias & Nogales, 2008			
Gallotia galloti	Wall lizard	P.F.	0.04	5	*Opuntia dillenii*	Valido & Nogales 1994			
Gallotia stehlini	Gran Canaria giant lizard	P.F.	0.5	9	*Phoenix canariensis*	González-Castro et al. 2012			
Gazella bennettii	Indian gazelle	N.F.	19	4	*Prosopis cineraria*	Dookia & Jakher 2007			
Geneta genetta	Common genet	P.F.	1.8	7	*Olea europea*	Rosalino et al. 2010			
Geospiza fortis	Medium ground finch	N.F.	0.03	4	*Memordica charantia*	Heleno et al. 2011			
Giraffa camelopardalis	Giraffe	N.F.	800	12	*Acacia erioloba*	Milton & Dean 2001			
Glossophaga soricina	Pallas's long-tongued bat	P.F.	0.01	2	*Coussapoa oligocephala*	Castro-Luna & Galindo-González 2012			
Gopheros polyphemus	Gopher tortoise	N.F.	5.5	10	*Geobalanus oblongifolius*	Birkhead et al. 2005			
Gorilla gorilla	Western gorilla	Fru.	136	25	*Cola lizae*	Tutin et al. 1991			
Gracilinanus agilis	Agile opossum	P.F.	0.02	2.5	*Psychotria barbiflora*	Lessa & da Costa 2010			
Helarctos malayanus	Sun bear	P.F.	44	16	*Canarium pilosum*	McConkey & Galetti 1999			
Hemideina crassidens	Wellington tree weta	N.F.	0.005	1	*Fuchsia excorticata*	King et al. 2011			

Sepecies	Common name	Diet	Body	Seed	Plant species	Reference	Max. DD	Mean DD	Distance Reference
Hemiphaga novaeseelandiae	New Zealand pigeon	Fru.	0.73	20	*Beilschmiedia tarairi*	McEwen 1978	1469	85	Wotton & Kelly 2012
Herpestes ichneumon	Egyptian mangoose	P.F.	2.6	5	*Pyrus bourgaeana*	Rosalino et al. 2010			
Hoplodactylus maculatus	New Zealand gecko	P.F.	0.01	3	*Coprosma propinqua*	Whitaker 1987	9	2	Wotton 2002
Hylobates lar	White-handed gibbons	Fru.	5.6	20	*Mangifera caloneura*	Phiphatsuwannachai et al. 2018	512	140	
Hylobates mulleri x agilis	Gibbons	Fru.	5.8	20.6	*Xanthophyllum amoenum*	McConkey et al. 2004	1250	220	
Hylobates pileatus	Pileated gibbon	Fru.	5.5	19		Kitamura et al. 2002			
Hypsipetes amaurotis	Brown-eared bulbul	Fru.	0.08	13.2	*Myrica rubra*	Noma & Yumoto 1997			
Iguana iguana	Green iguana	N.F.	3.1	9.2	*Protium sp.*	de Moura et al. 2015			
Irena puella	Asian fairy-bluebird	P.F.	0.06	5	*Dysoxylum binectariferum*	Seith & Howe 2012			
Kinosternon hirtipes	Rough-footed Mud Turtle	N.F.	0.21	5	*Prosopis glandulosa*	Platt et al. 2016			
Lagothrix flavicauda	Yellow tailed woolly monkey	P.F.	8.2	22	*Anomospermum sp.*	Hernandez & Shanee, pers. comm.			
Lagothrix lagothricha	Woolly monkey	Fru.	7.5	20	*Tontelea sp.*	Ramirez et al. 2014	1466	345	
Leontopithecus chrysomelas	Golden-headed lion tamarin	P.F.	0.6	15	*Cheiloclinium cognatum*	Cardoso et al. 2011	781		
Leontopithecus rosalia	Golden lion tamarin	P.F.	0.62	9.5	*Abuta sellowiana*	Dietz et al. 1997	858	101	
Lepus europaeus	European Brown Hares	N.F.	3.9	3	*Vicia sativa*	Panter & Dolman 2012			
Lepus saxatilis	Scrub hare	N.F.	3	2	*Galenia fruticosa*	Milton & Dean 2001			
Liolaemus pictus	Chilean lizard	P.F.	0.2	3	*Nertera granadensis*	Willson et al. 1996			
Liopholis guthega	Guthega skink	N.F.	0.015	3	*Melicytus dentatus*	Atkins et al. 2018			
Lithodoras dorsalis	Bottom dwelling catfish	P.F.	15	22	*Astrocaryum jauari*	Kubitzki & Kubitzki 1994			
Lophocebus albigena	Gray cheeked mangabey	P.F.	3.6	18	*Vitex grandifolia*	Poulsen et al. 2001		42	
Loxodonta africana	African Elephant	P.F.	3246	48.7	*Balanites wilsoniana,*	Lieberman et al. 1997	57000	3500	Blake et al. 2009
Lumbricus terrestris	European earthworm	N.F.	0.004	5	*Ambrosia trifida*	Regnier et al. 2008			

Sepecies	Common name	Diet	Body	Seed	Plant species	Reference	Max. DD	Mean DD	Distance Reference
Lycalopex gymnocercus	Pampas fox	P.F.	6.5	15	*Geoffroea decorticans*	Varela et al. 2008			
Macaca cyclopis	Formosan rock macaques	P.F.	5.7	4.9		Chen 2002			
Macaca fascicularis	Long tailed macaque	Fru.	5.3	10	*Leuconotis griffithii*	Lucas & Corlett 1998			
Macaca fuscata	Japanese macaques	Fru.	10	11.8	*Diospyros lotus*	Tsuji et al. 2011a	634		
Macaca leonina	Pigtailed macaque	P.F.	6.4	13.6	*Garcinia benthamii*	Albert et al. 2013			
Macaca mulatta	Rhesus macaques	P.F.	7.75	17.1		Tsuji et al. 2013	774	117	
Macaca nemestrina	Pigtail macaque	P.F.	6.5	16.7	*Platymitra macrocarpa*	Kitamura et al. 2002			
Macroclemys temminckii	Alligator snapping turtle	P.F.	114	21	*Carya illinoensis*	Sloan et al. 1996			
Manacus manacus	White-beaded manakin	Fru.	0.014	9	*Eugenia sulcata*	Cestari & Pizo 2013			
Mandrillus leucophaeus	Drill	P.F.	16	20	*Trichoscypha acuminata*	Astaras & Waltert 2010			
Marmosops incanus	Gray slender mouse opossum	P.F.	0.06	2.5	*Psychotria barbiflora*	Lessa & da Costa 2010			
Marmosops paulensis	Brazilian slender opossum	P.F.	0.04	2.2	*Psidium cattleyanum*	Leiner & Silva 2007			
Marten flavigula	Yellow-throated marten	P.F.	3.4	8	*Diospyros lotus*	Zhou et al. 2008b			
Martes americana	American marten	N.F.	0.9	2	*Rubus spectabilis*	Hickey et al. 1999			
Martes foina	Stone marten	P.F.	1.7	16	*Prunus communis*	Bakaloudis et al. 2012	1117	447	Herrera et al. 2016
Martes martes	Pine marten	P.F.	1.7	10	*Prunus domestica*	Schaumann & Heinken 2002	2846	408	
Martes melampus	Japanese marten	P.F.	1.6	11	*Torreya nucifera*	Koike & Masaki 2019	1500	770	
Mazama americana	Red brocket deer	N.F.	33	4	*Rubiaceae*	Bodmer 1991			
Megalaima faiostricta	Green eared barbet	Fru.	0.096	9.5	*Litsea monopetala*	Sankamethawee et al. 2011			
Megalaima nuchalis	Taiwan barbets	Fru.	0.09	10	*Machilus japonica*	Chang et al. 2012			
Meles meles	Badger	P.F.	12	7	*Olea europea*	Rosalino et al. 2010			
Melogale moschata	Ferret-badgers	P.F.	0.9	8	*Prunus salicina*	Zhou et al. 2008a			
Melursus ursinus	Sloth bear	P.F.	86.5	30	*Mangifera indica*	Bargali et al. 2004			

Sepecies	Common name	Diet	Body	Seed	Plant species	Reference	Max. DD	Mean DD	Distance Reference
Metachirus nudicaudatus	Brown four-eyed opossums	N.F.	0.4	5	*Amaioua guianensis*	Lessa & da Costa 2010			
Micoureus paraguayanus	Tate's wolly mouse opossum	P.F.	0.11	5	*Amaioua guianensis*	Lessa & da Costa 2010			
Microcebus jollyae	Jolly's mouse lemur	P.F.	0.06	5	*Coffea millotii*	Ramananjato et al. 2020			
Microcebus murinus	Gray mouse lemur	P.F.	0.06	6	*Erythroxylon sp.*	Lahann 2007			
Microcebus rufus	Brown mouse lemur	P.F.	0.04	12	*Tambourissa thouvenotii*	Ramananjato et al. 2020			
Microlophus delanonis	Lava lizard	P.F.	0.23	6	*Bursera graveolens*	Hervías-Parejo et al. 2019			
Mitu salvini	Salvin's curassow	N.F.	3	7	*Protium sagotianum*	Santamaria & Franco 2002, Jiménez pers. comm.			
Monticola solitarius	Blue rockthrush	N.F.	0.06	7	*Neolitsea sp.*	Emura et al. 2012			
Muntiacus muntjak	Muntjak	N.F.	20	3.5	*Triticum spp.*	Panter & Dolman 2012			
Mustela itatsi	Japanese weasel	P.F.	0.4	6	*Diospyros kaki*	Tsuji et al. 2011b			
Myadestes melanops	Black-faced solitaires	Fru.	0.03	2.4	*Wintheringia coccoloboides*	Murray 1988	370	48	
Myiopsitta monachus	Monk parakeet	N.F.	0.1	0.8	*Plantago major*	Blanco et al. 1916			
Myloplus tiete	Pacu-manteiga	P.F.	0.14	7	*Byrsonima laxiflora*	Correa et al. 2015			
Myloplus torquatus	Pacu-prata	P.F.	0.86	10.7	*Calophyllum sp.*	Correa et al. 2015			
Mylossoma duriventre	Silver dollar fish	P.F.	1	2.5	*Ilex sp.*	Correa et al. 2015			
Nasalis larvatus	Proboscis monkey	N.F.	14.5	5	*Bridelia stipularis*	Thiry et al. 2019			
Nasua nasua	Coati	P.F.	4.5	17.2	*Cryptocarya aschersoniana*	Alves-Costa & Eterovick 2007			
Nestor notabilis	New Zealand kea	P.F.	0.87	6	*Podocarpus nivalis*	Young et al. 2012			
Noctilio albiventris	lesser bulldog bat	P.F.	0.03	2.5	*Solanum bicolor*	Aranguren et al. 2011			
Nomascus concolor	Black crested gibbon	P.F.	5	12.7	*Symplocos ramosissima*	Fan et al. 2008	425	179	Hai et al. 2018
Nyctereutes procyonoides	Racoon dog	P.F.	4.1	11	*Torreya nucifera*	Koike & Masaki 2019	938	253	
Odocoileus virginianus	White-tailed deer	N.F.	56.5	7	*Prunus serotina*	Williams et al. 2008	3730	500	Vellend et al. 2003

Sepecies	Common name	Diet	Body	Seed	Plant species	Reference	Max. DD	Mean DD	Distance Reference
Orycteropus afer	Aardvark	N.F.	53	4	*Augea capensis*	Milton & Dean 2001			
Oryctolagus cuniculus	Rabbit	N.F.	2	4.5	*Crataegus monogyna*	Mancilla-Leyton et al. 2013			
Oryzomys galapagoensis	Galápagos rice rat	N.F.	0.06	5	*Cordia lutea*	Heleno et al. 2011			
Ovibos moschatus	Muskox	N.F.	260	2	*Polygonum sp.*	Bruun et al. 2008			
Ovis aries	Domestic sheep	N.F.	87	4	*Acacia thunbergii*	Horn et al. 2013			
Paguma larvata	Masked palm civet	P.F.	4.3	8	*Prunus salicina*	Zhou et al. 2008c			
Pan paniscus	Bonobo	Fru.	39	20	*Omphalocarpum elatum*	Trolliet et al. 2016	2995	1183	Beaune et al. 2013
Pan troglodytes	Chimpanzee	Fru.	42	33	*Beilschmiedia mani*	Gautier-Hion et al. 1985			
Papio anubis	Olive baboon	P.F.	19.5	21.8	*Vitex doniana*	Kunz et al. 2008			
Papio ursinus	Chacma baboons	P.F.	12	6	*Grewia occidentalis*	Slater & du Toit 2002			
Paradisaea raggiana	Bird of paradise	Fru.	0.25	13	*Dysoxylum pettigrewianum*	Dumbacher & Beehler 1996			
Paradoxurus hermaphroditus	Common palm civet	P.F.	3.2	17	*Arenga pinnata*	Subrata & Syahbudin 2016			
Penelope superciliaris	Rusty-margined guan	Fru.	1.05	15	*Pseudina frutescens*	Pizo 2004			
Perdix perdix	Grey partridge	N.F.	0.38	1.5	*Plantago aviculare*	Orłowski & Czarnecka 2012			
Phainoptila melanoxantha	Black-and-yellow silky-flycatcher	P.F.	0.06	2.4	*Wintheringia coccoloboides*	Murray 1988	510	50	
Pharomachrus mocinno	Resplandescent quetzal	Fru.	0.21	17	*Licaria excelsa*	Avila et al. 1996			
Philander frenata	Gray four-eyed opossums	N.F.	0.32	5	*Monstera adansoni*	Cáceres 2004			
Phoenicurus ochruros	Black redstart	N.F.	0.02	3	*Viburnum lantana*	Czarnecka et al. 2012			
Piaractus brachypomus	Cachama blanca	P.F.	25	12	*Bactris bidentula*	Anderson et al. 2009			
Piaractus mesopotamicus	Pacu	P.F.	20	19.6	*Couepia uiti*	Correa et al. 2015			
Pimelia costata	Tenebrionid beetle	N.F.	0.007	0.2	*Cytinus hypocistis*	de Vega et al. 2011			
Pithecia pithecia	White-faced sakis	N.F.	2.1	2	*Cecropia sp.*	Norconck et al. 1998			

Sepecies	Common name	Diet	Body	Seed	Plant species	Reference	Max. DD	Mean DD	Distance Reference
Platyrrhinus dorsalis	Thomas's broad-nosed bat	Fru.	0.03	1.2	*Cecropia peltata*	Zapata-Mesa et al. 2017			
Platyrrhinus lineatus	White-lined broad-nosed bat	Fru.	0.02	1.3	*Solanum mauritianum*	Silveira et al. 2011			
Platyrrhinus nitelinea	Broad-nosed bat	Fru.	0.03	1.3	*Cecropia obtusifolia*	Zapata-Mesa et al. 2017			
Platyspiza crassirostris	Vegetarian finch	N.F.	0.04	0.6	*Miconia robinsoniana*	Heleno et al. 2011			
Platysternon megacephalum	Big-headed turtle	N.F.	0.39	10	*Machilus thunbergii*	Sung et al. 2016			
Podarcis lilfordi	Lilford's wall lizard	P.F.	0.01	4.5	*Phillyrea latifolia*	Perez-Mellado & Traveset 1999			
Pongo pygmaeus	Orangutan	Fru.	57.5	18	*Diospyros areolata*	Tarszisz et al. 2018			
Pottos flavus	Kinkajou	P.F.	3	14	*Astrocaryum standleyanum*	Kays 1999			
Procyon cancrivorus	Raccoon	P.F.	5	10	*Allagoptera arenari*	Gatti et al. 2006			
Propithecus diadema	Diademed sifaka	N.F.	6.5	2	*Sofanum auricutatum*	Dew & Wright 1998			
Prosthemadera novaeseelandiae	Tuis	P.F.	0.09	9	*Alectryon excelsus*	Williams & Karl 1996			
Pseudalopex culpaeus	Culpeo fox	P.F.	8.6	6	*Schinus polygamus*	Silva et al. 2005			
Pseudalopex gymnocercus	Pampa fox	P.F.	5.2	20	*Syagrus romanzoffiana*	Varela & Bucher 2006			
Psittacara hockingi	Hocking's parakeet	N.F.	0.18	2	*Maclura tinctoria*	Blanco et al. 1916			
Pterodoras granulosus	Granulated katfish	P.F.	6.5	25	*Syagrus romazoffianum*	Conceicao et al. 1994			
Pteroglossus castanotis	Chestnut-eared aracari	P.F.	0.26	12	*Ocotea oblonga*	Rosado & Linares 2017			
Pteropus alecto	Black flying fox	Fru.	0.61	8	*Carpentaria acuminata*	Palmer et al. 2000			
Pteropus conspicillatus	Spectacled flying fox	P.F.	0.7	3.2	*Psidium guajava*	Richards 1990			
Pteropus dasymallus	Orii's flying-fox	Fru.	0.45	1.4	*Idesia polycarpa*	Nakamoto et al. 2009	1833	150	
Pteropus giganteus	Indian ying fox	Fru.	1.2	7.7	*Areca catechu*	Gulraiz et al. 2016			
Pteropus niger	Mauritian flying fox	Fru.	0.46	1	*Ficus reflexa*	Nyhagen et. 2005, Olesky 2015			
Pteropus rufus	Madagascaran flying fox	Fru.	0.75	9.6	*Uapaca sp.*	Bollen & Elsacker 2002			
Pteropus tonganus	Insular flying fox	Fru.	0.56	5		McConkey & Drake 2006			

Sepecies	Common name	Diet	Body	Seed	Plant species	Reference	Max. DD	Mean DD	Distance Reference
Pteropus voeltzkowi	Pemba flying fox	Fru.	0.55	1	*Ficus spp.*	Entwistle & Corp 1997			
Ptilinopus pulchellus	Beautiful fruit dove	Fru.	0.07	11	*Myristica sp.*	Dumbacher & Beehler 1996			
Ptilinopus purpuratus	Grey-green fruit dove	Fru.	0.09	8	*Cyclophyllum barbatum*	Spotswood et al. 2012			
Pycnonotus barbatus	Common bulbul	P.F.	0.04	6	*Prunus africana*	Farwig et al. 2006			
Pycnonotus cafer	Red-vented bulbul	P.F.	0.04	8	*Cyclophyllum barbatum*	Spotswood et al. 2012			
Pycnonotus flaviventris	Black-crested bulbu	P.F.	0.03	6.5	*Magnolia baillonii*	Khamcha et al. 2014	141	25	
Pycnonotus jocosus	Red-whiskered bulbul	Fru.	0.04	13	*Litsea glutinosa*	Kitamura et al. 2002	1400	131	Weir & Corlett 2007
Ramphastos dicolorus	Toco tucan	P.F.	0.65	20	*Virola gardneri*	Galleti et al. 2000			
Ramphastos toco	Toco toucan	P.F.	0.62	8	*Vitex cymosa*	Ragusa-Netto 2006	800	144	
Ramphocelus costaricensis	Cherrie's tanager	P.F.	0.03	3	*Lantana camara*	Lindell et al. 2013			
Rattus rattus	Black rat	N.F.	0.14	4	*Bursera graveolens*	Heleno et al. 2011			
Rhabdotorrhinus waldeni	Dulungan hornbill	Fru.	0.8	22	*Pometia pinnata*	Hamann & Curio 1999			
Rhea americana	Greater rhea	P.F.	23.5	30	*Spondias tuberosa*	de Azevedo et al. 2013			
Rhinoceros unicornis	Greater one-horned rhinoceros	N.F.	2250	23	*Spondias pinnata*	Srinhara et al. 2016			
Rhinoclemmys funerea	Brown wood turtle	P.F.	4.5	18	*Spondias mombin*	Moll & Jansen 1995			
Rousettus aegyptiacus	Egyptian fruit-bat	Fru.	0.12	11	*Eriobotrya japonica*	Korine et al. 1999			
Ruwenzorornis johnstoni	Ruwenzori turaco	Fru.	0.24	13	*Ekebergica capensis*	Sun et al. 1997			
Saguinus fuscicollis	Saddleback tamarin	P.F.	0.45	13.5	*Buchenavia oxycarpa*	Knogge & Haymann 2003	656	239	
Saguinus geoffroyi	Panamanian tamarin	P.F.	0.50	16		Garber & Kitron 1997			
Saguinus leucopus	White-footed tamarin	Fru.	0.46	8	*Diospyros vestita*	de Luna et al. 2016	529	206	
Saguinus midas	Black-handed tamarins	P.F.	0.55	10		Oliveira & Ferrari 2000			
Saguinus mystax	Moustached tamarin	P.F.	0.69	13.1	*Buchenavia oxycarpa*	Knogge & Haymann 2003			
Saimiri sciureus	Squirrel monkey	P.F.	0.9	9.25	*Tapirira guianensis*	Oliveira-Silva et al. 2018			
Saltator maximus	Buff-throated saltator	P.F.	0.04	3	*Cissampelos pareira*	Lindell et al. 2013			

Sepecies	Common name	Diet	Body	Seed	Plant species	Reference	Max. DD	Mean DD	Distance Reference
Sapajus apella (syn. Cebus apella)	Brown capuchin	P.F.	3.05	15	*Micropholis* melinoniana	Guillotin et al. 1994		355	
Sapajus libidinosus	Capuchin monkey	P.F.	2.4	8	*Copaifera cf. langsdorfii*	Moura & McConkey 2007			
Sapajus xanthosternos	Yellow-breasted capuchins	P.F.	3	21	*Tetrastylidium grandifolium*	Canale et al. 2016		330	
Semnornis frantzii	Prong Billed Barbet	Fru.	0.06	2.4	*Wintheringia coccoloboides*	Murray 1988		40	
Sialia sialis	Eastern Bluebirds	P.F.	0.03	3.5	*Juniperus virginiana*	Weinkam et al. 2017			
Sphenurus formosae	Red-capped green pigeon	Fru.	0.24	16	*Symplocos prunifolia*	Noma & Yumoto 1997			
Steatornis carpipensis	Oilbird	Fru.	0.4	29	*Rhodostemonodaphne grandis*	Stevenson et al. 2017	47584	10085	This study
Sturnira erythromos	Little yellow-shouldered bat	Fru.	0.02	6	*Celtis iguanaeus*	Sanchez et al. 2012			
Sturnira lilium	Little yellow-shouldered bat	Fru.	0.02	6	*Celtis iguanaeus*	Sanchez et al. 2012			
Sturnus vulgaris	Starling	P.F.	0.08	1.5	*Sambucus nigra*	Williams & Karl 1996			
Sus scrofa	Wild boar	N.F.	135	28	*Mangifera sp.*	Heydon 1994			
Sylvia atricapilla	Blackcaps	P.F.	0.02	7	*Olea europea*	Telleria et al. 2013			
Sylvicapra grimmia	Grey duiker	P.F.	16	9	*Diospyros lycioides*	Milton & Dean 2001			
Sylvilagus audubonii	Desert cottontail	N.F.	1.14	4	*Juniperus osteosperma*	Schupp et al. 1997			
Sylvilagus bachmani	Rabbit	N.F.	0.7	1.5	*Lythrum hyssopifolia*	Zedler & Black 1992			
Tangara icterocephala	Silver-throated tanager	Fru.	0.02	3.1	*Croton draco*	Lindell et al. 2013			
Tapirus bairdii	Central American tapir	P.F.	250	40	*Licania platypus*	Naranjo 2009			
Tapirus indicus	Malayan tapir	P.F.	318.3	26	*Durio zibethinus*	Campos-Arceiz et al. 2012	3289	1102	Campos-Arceiz et al. 2012
Tapirus pinchaque	Mountain tapir	P.F.	200	8	*Prunus serotina*	Downer 2001			

Sepecies	Common name	Diet	Body	Seed	Plant species	Reference	Max. DD	Mean DD	Distance Reference
Tapirus terrestris	Lowland tapir	P.F.	225	40	*Mauritia flexuosa*	Fragoso & Huffman 2000			
Tauraco schuettii	Black billed turaco	Fru.	0.23	17	*Strombosciа scheffleri*	Sun et al. 1997			
Taurotragus oryx	Eland	N.F.	563	9	*Acacia caffra*	Slater & du Toit 2002			
Tayassu pecari	White-lipped peccary	N.F.	33.2	20	*Syagrus romazoffiana*	Keuroghlian & Eaton 2008			
Tayassu pecari	Collared peccary	N.F.	22	5	*Cucurbita foetidissima*	Platt et al. 2014			
Teius teyou	Four-toed tegu	P.F.	0.03	6	*Ziziphus mistol*	Varela & Bucher 2002b			
Terrapene carolina	Florida box turtle	P.F.	0.8	10	*Serenoa repens*	Liu et al. 2004			
Thectocercus acuticaudatus	Blue-crowned conure or parakeet	N.F.	0.17	2	*Pilosocereus pachycladus*	Blanco et al. 1916			
Thraupis sayaca	Sayaca tanager	Fru.	0.03	5	*Chrysophyllum marginatum*	Gianini 1999			
Timon lepidus	Eyed lizard	P.F.	0.45	4.5	*Tamus communis*	Piazzon et al. 2012	170	40	
Tolypeutes matacus	Three banded armadillo	N.F.	1.5	9	*Zyzyphys mistol*	Bolkovic et al. 1995			
Trachypithecus auratus	Javan lutungs	N.F.	7	4.5	*Celtis phillipensis*	Tsuji et al. 2016	299	108	
Tragelaphus strepsiceros	Greater kudu	N.F.	198	12	*Acacia erioloba*	Milton & Dean 2001			
Tragulus napu	Greater mouse-deer	N.F.	7	2	*Polyalthia sumatrana*	Heydon 1994			
Trichosurus vulpecula	Brushtail possum	N.F.	2.34	12	*Elaeocarpus dentatus*	Cowan 1990			
Trogon melanocephalus	Black-headed trogon	Fru.	0.08	8	*Ocotea veraguensis*	Riehl & Adelson 2008			
Turdus albicollis	White- necked Thrush	P.F.	0.05	12	*Cabralea canjerana*	de Castro et al. 2012			
Turdus amaurocalinus	Creamy-bellied thrush	Fru.	0.06	5	*Eugenia uniflora*	Gianini 1999			
Turdus falcklandii	Austral thrush	P.F.	0.08	6	*Schinus polygamus*	Reid & Armesto 2011			
Turdus flavipes	Yellow-legged thrush	P.F.	0.06	12	*Euterpe edulis*	de Castro et al. 2012			
Turdus grayi	Clay-colored thrush	P.F.	0.07	3	*Siparuna pauciflora*	Lindell et al. 2013			
Turdus iliacu	Redwing	P.F.	0.06	3	*Empetrum nigrum*	Guitian et al. 1994			
Turdus merula	Eurasian blackbird	P.F.	0.11	9	*Alectryon excelsus*	Williams & Karl 1996	300		Jordano et al. 2007
Turdus migratorius	American robin	Fru.	0.08	7	*Prunus serotina*	Witmer 1996			

Sepecies	Common name	Diet	Body	Seed	Plant species	Reference	Max. DD	Mean DD	Distance Reference
Turdus naumanni	Naumann's thrush	P.F.	0.08	5	*Viburnum dilatatum*	Nishi & Tsuyuzaki 2004			
Turdus pallidus	Pale thrush	P.F.	0.08	6.7	*Ardisia sieboldii*	Noma & Yumoto 1997			
Turdus philomelos	Song thrushes	P.F.	0.07	6.6	*Berberis glaucocarpa*	Wyman & Kelly 2017			
Turdus rufiventris	Rufous-bellied thrush	Fru.	0.06	6	*Celtis iguanaeus*	Blendinger et al. 2015			
Ursus americanus	American black bear	P.F.	120	8	*Prunus virginiana*	Enders & Vander Wall 2012			
Ursus thibetanus	Japanese black bear	P.F.	104	25	*Juglans mandshurica*	Takahashi et al. 2008	22000	1250	Koike & Masaki 2019
Vampyressa pusilla	Southern yellow-eared bat	Fru.	0.02	1.3	*Solanum mauritianum*	Silveira et al. 2011			
Varanus olivaceus	Gray's monitor	P.F.	6.7	20	*Canarium hirsutum*	Auffenberg 1988			
Varecia rubra	Red- ruffed lemur	Fru.	3.9	24.7	*Ampelosicyos humblotii*	Razafindratsima & Martinez 2012	568	48	
Varecia variegata	Black and white ruffed lemur	Fru.	3.6	22	*Urticaceae (liana)*	Moses & Semple 2011, Dew & Wright 1998,	630	117	Razafindratsima et al. 2014
Vulpes corsac	Corsac fox	P.F.	2.4	11	*Amygdalus pedunculata*	Murdoch et al. 2009			
Vulpes vulpes	Red fox	P.F.	5.5	11	*Amygdalus pedunculata*	Murdoch et al. 2009	2846	1321	
Xenohyla truncata	Fruit eating frog	P.F.	0.11	6	*Maytenus obtusifolia*	da Silva & Britto-Pereira 2006			
Zonotrichia capensis	Rufous-collared sparrow	N.F.	0.02	6	*Schinus polygamus*	Reid & Armesto 2011			
Zosterops japonicus	Japanese white-eye	P.F.	0.01	6.2	*Cleyera japonica*	Noma & Yumoto 1997			
Zosterops lateralis	Silvereye	Fru.	0.01	6.6	*Berberis glaucocarpa*	Wyman & Kelly 2017			

Revised studies

Abedi-Lartey, M., Dechmann, D. K., Wikelski, M., Scharf, A. K., & Fahr, J. (2016). Long-distance seed dispersal by straw-coloured fruit bats varies by season and landscape. *Global Ecology and Conservation*, *7*, 12-24.

Agmen, F. L., Chapman, H. M., & Bawuro, M. (2010). Seed dispersal by tantalus monkeys (*Chlorocebus tantalus tantalus*) in a Nigerian montane forest. *African Journal of Ecology, 48*(4), 1123-1128.

Albert, A., Hambuckers, A., Culot, L., Savini, T., & Huynen, M. C. (2013). Frugivory and seed dispersal by northern pigtailed macaques (*Macaca leonina*), in Thailand. *International Journal of Primatology*, *34*(1), 170-193.

Alves-Costa, C. P., & Eterovick, P. C. (2007). Seed dispersal services by coatis (*Nasua nasua,* Procyonidae) and their redundancy with other frugivores in southeastern Brazil. *Acta Oecologica*, *32*(1), 77-92.

Amico, G. C., Rodríguez-Cabal, M. A., & Aizen, M. A. (2009). The potential key seed-dispersing role of the arboreal marsupial *Dromiciops gliroides*. *Acta oecologica*, *35*(1), 8-13.

Anderson, J. T., Rojas, J. S., & Flecker, A. S. (2009). High-quality seed dispersal by fruit-eating fishes in Amazonian floodplain habitats. *Oecologia*, *161*(2), 279-290.

Anderson, J. T., Nuttle, T., Saldaña Rojas, J. S., Pendergast, T. H., & Flecker, A. S. (2011). Extremely long-distance seed dispersal by an overfished Amazonian frugivore. *Proceedings of the Royal Society B: Biological Sciences*, *278*(1723), 3329-3335.

Aranguren, C. I., González-Carcacía, J. A., Martínez, H., & Nassar, J. M. (2011). *Noctilio albiventris* (Noctilionidae), a potential seed disperser in disturbed tropical dry forest habitats. *Acta Chiropterologica*, *13*(1), 189-194.

Astaras, C., & Waltert, M. (2010). What does seed handling by the drill tell us about the ecological services of terrestrial cercopithecines in African forests?. *Animal conservation*, *13*(6), 568-578.

Atkins, Z. S., Clemann, N., Schroder, M., Chapple, D. G., Davis, N. E., Robinson, W. A., ... & Robert, K. A. (2018). Consistent temporal variation in the diet of an endangered alpine lizard across two south-eastern Australian sky-islands. *Austral Ecology*, *43*(3), 339-351.

Auffenberg, W. (1988). Gray's monitor lizard. University Press of Florida.

Avila-H. M. L., V. Hugo Hernandez O., & Verlarde, E. (1996). The Diet of Resplendent Quetzal (*Pharomachrus Moncinno mocinno*: Trogonidae) in a Mexican Cloud Forest. *Biotropica*, 720-727.

Baiao, S. A., Correia, F. B., & Ferrari, S. F. (2015). Dietary differences have contrasting effects on the seed dispersal potential of the titi monkey *Callicebus coimbrai* in north-eastern Brazil. *Journal of Tropical Ecology*, *31*(2), 175-181.

Bakaloudis, D. E., Vlachos, C. G., Papakosta, M. A., Bontzorlos, V. A., & Chatzinikos, E. N. (2012). Diet composition and feeding strategies of the stone marten (*Martes foina*) in a typical Mediterranean ecosystem. *The Scientific World Journal*, *2012*.

Bargali, H. S., Akhtar, N., & Chauhan, N. P. S. (2004). Feeding ecology of sloth bears in a disturbed area in central India. *Ursus, 15*(2), 212-217.

Beaune, D., Bretagnolle, F., Bollache, L., Bourson, C., Hohmann, G., & Fruth, B. (2013). Ecological services performed by the bonobo (*Pan paniscus*): seed dispersal effectiveness in tropical forest. *Journal of Tropical Ecology, 29*(5), 367-380.

Benítez-Malvido, J., González-Di Pierro, A. M., Lombera, R., Guillén, S., & Estrada, A. (2014). Seed source, seed traits, and frugivore habits: Implications for dispersal quality of two sympatric primates. *American Journal of Botany*, *101*(6), 970-978.

Birkhead, R. D., Guyer, C., Hermann, S. M., & Michener, W. K. (2005). Patterns of folivory and seed ingestion by gopher tortoises (*Gopherus polyphemus*) in a southeastern pine savanna. *The American midland naturalist, 154*(1), 143-151.

Birkinshaw, C. (2001). Fruit characteristics of species dispersed by the black lemur (*Eulemur macaco*) in the Lokobe Forest, Madagascar. *Biotropica*, *33*(3), 478-486.

Blake, S., Deem, S. L., Mossimbo, E., Maisels, F., & Walsh, P. (2009). Forest elephants: tree planters of the Congo. *Biotropica*, *41*(4), 459-468.

Blake, S., Wikelski, M., Cabrera, F., Guezou, A., Silva, M., Sadeghayobi, E., Yackulic, C.B. & Jaramillo, P. (2012). Seed dispersal by Galápagos tortoises. *Journal of Biogeography, 39*(11), 1961-1972.

Blake, S., Guézou, A., Deem, S. L., Yackulic, C. B., & Cabrera, F. (2015). The dominance of introduced plant species in the diets of migratory Galapagos tortoises increases with elevation on a human-occupied island. Biotropica, 47(2), 246-258.

Blanco, G., Bravo, C., Pacifico, E. C., Chamorro, D., Speziale, K. L., Lambertucci, S. A., ... & Tella, J. L. (2016). Internal seed dispersal by parrots: an overview of a neglected mutualism. *PeerJ*, *4*, e1688.

Blázquez, M. C., & Rodríguez-Estrella, R. (2007). Microhabitat selection in diet and trophic ecology of a spiny-tailed iguana *Ctenosaura hemilopha*. *Biotropica, 39*(4), 496-501.

Blendinger, P. G., Giannini, N. P., Zampini, I. C., Ordoñez, R., Torres, S., Sayago, J. E., ... & Isla, M. I. (2015). Nutrients in fruits as determinants of resource tracking by birds. *Ibis*, *157*(3), 480-495.

Bobadilla, S. Y., Benitez, V. V., & Guichón, M. L. (2016). Asiatic *Callosciurus squirrels* as seed dispersers of exotic plants in the Pampas. *Current Zoology*, *62*(3), 215-219.

Bodmer, R. E. (1991). Strategies of seed dispersal and seed predation in Amazonian ungulates. *Biotropica*, 255-261.

Böhning-Gaese, K., Gaese, B. H., & Rabemanantsoa, S. B. (1999). Importance of primary and secondary seed dispersal in the Malagasy tree *Commiphora guillaumini*. Ecology, 80(3), 821-832.

Bolković, M. L., Caziani, S. M., & Protomastro, J. J. (1995). Food habits of the three-banded armadillo (Xenarthra: Dasypodidae) in the dry Chaco, Argentina. *Journal of Mammalogy*, *76*(4), 1199-1204.

Bollen, A., & Van Elsacker, L. (2002). Feeding ecology of *Pteropus rufus* (Pteropodidae) in the littoral forest of Sainte Luce, SE Madagascar. *Acta Chiropterologica*, *4*(1), 33-47.

Boubli, J. P. (1999). Feeding ecology of black-headed uacaris (*Cacajao melanocephalus melanocephalus*) in Pico da Neblina National Park, Brazil. *International Journal of Primatology*, *20*(5), 719-749.

Brochet, A. L., Guillemain, M., Fritz, H., Gauthier-Clerc, M., & Green, A. J. (2009). The role of migratory ducks in the long-distance dispersal of native plants and the spread of exotic plants in Europe. *Ecography*, *32*(6), 919-928.

Bruun, H. H., Lundgren, R., & Philipp, M. (2008). Enhancement of local species richness in tundra by seed dispersal through guts of muskox and barnacle goose. *Oecologia*, *155*(1), 101-110.

Bueno, A., & Motta-Junior, J. C. (2009). Feeding habits of the maned wolf, *Chrysocyon brachyurus* (Carnivora: Canidae), in southeast Brazil. *Studies on Neotropical Fauna and Environment*, *44*(2), 67-75.

Cáceres, N. C. (2002). Food habits and seed dispersal by the white-eared opossum, *Didelphis albiventris*, in southern Brazil. *Studies on Neotropical Fauna and Environment*, *37*(2), 97-104.

Cáceres, N. C. (2004). Diet of three didelphid marsupials (Mammalia, Didelphimorphia) in southern Brazil. *Mammalian Biology*, *69*(6), 430-433.

Calviño-Cancela, M., R. Dunn, R., Van Etten, E. J., & B. Lamont, B. (2006). Emus as non-standard seed dispersers and their potential for long-distance dispersal. *Ecography*, *29*(4), 632-640.

Campos-Arceiz, A., Traeholt, C., Jaffar, R., Santamaria, L., & Corlett, R. T. (2012). Asian tapirs are no elephants when it comes to seed dispersal. *Biotropica, 44*(2), 220-227.

Canale, G. R., Suscke, P., Rocha-Santos, L., São Bernardo, C. S., Kierulff, M. C. M., & Chivers, D. J. (2016). Seed dispersal of threatened tree species by a critically endangered primate in a Brazilian Hotspot. *Folia Primatologica*, *87*(3), 123-140.

Cardoso, N. A., Le Pendu, Y., Lapenta, M. J., & Raboy, B. E. (2011). Frugivory patterns and seed dispersal by golden-headed lion tamarins (*Leontopithecus chrysomelas*) in Una Biological Reserve, Bahia, Brazil. *Mammalia, 75*(4), 327-337.

Carpenter, J. K., O'Donnell, C. F., Moltchanova, E., & Kelly, D. (2019). Long seed dispersal distances by an inquisitive flightless rail (*Gallirallus australis*) are reduced by interaction with humans. *Royal Society Open Science, 6*(8), 190397.

Casella, J., & Cáceres, N. C. (2006). Diet of four small mammal species from Atlantic forest patches in South Brazil. *Neotropical Biology and Conservation*, *1*(1), 5-11.

Castro-Luna, A. A., & Galindo-González, J. (2012). Seed dispersal by phyllostomid bats in two contrasting vegetation types in a Mesoamerican reserve. *Acta chiropterologica*, *14*(1), 133-142.

Cestari, C., & Pizo, M. A. (2013). Frugivory by the White-bearded Manakin (*Manacus manacus*, Pipridae) in restinga forest, an ecosystem associated to the Atlantic forest. *Biota Neotropica*, *13*(2), 345-350.

Chang, S. Y., Lee, Y. F., Kuo, Y. M., & Chen, J. H. (2012). Frugivory by Taiwan Barbets (*Megalaima nuchalis*) and the effects of deinhibition and scarification on seed germination. *Canadian Journal of Zoology*, *90*(5), 640-650.

Chapman, H. M., Goldson, S. L., & Beck, J. (2010). Postdispersal removal and germination of seed dispersed by *Cercopithecus nictitans* in a West African montane forest. *Folia primatologica*, *81*(1), 41-50.

Chen, C. E. (2002). Seed dispersal by Formosan macaques (*Macaca cyclopis*) in the Fusan Experimental Forest of Taiwan. PhD dissertation: National Taiwan University.

Chiarello, A. G. (1998). Diet of the Atlantic forest maned sloth *Bradypus torquatus* (Xenarthra: Bradypodidae). *Journal of Zoology*, *246*(1), 11-19.

Conceiçao de Souza-Stevaux, M., Negrelle, R. R., & Citadini-Zanette, V. (1994). Seed dispersal by the fish *Pterodoras granulosus* in the Paraná River Basin, Brazil. *Journal of Tropical Ecology, 10*(4), 621-626.

Correa, S. B., Araujo, J. K., Penha, J. M., da Cunha, C. N., Stevenson, P. R., & Anderson, J. T. (2015). Overfishing disrupts an ancient mutualism between frugivorous fishes and plants in Neotropical wetlands. *Biological Conservation*, *191*, 159-167.

Cosyns, E., Claerbout, S., Lamoot, I., & Hoffmann, M. (2005). Endozoochorous seed dispersal by cattle and horse in a spatially heterogeneous landscape. *Plant ecology*, *178*(2), 149-162.

Couvreur, M., Cosyns, E., Hermy, M., & Hoffmann, M. (2005). Complementarity of epi-and endozoochory of plant seeds by free ranging donkeys. *Ecography*, *28*(1), 37-48.

Cowan, P. E. (1990). Fruits, seeds, and flowers in the diet of brushtail possums, *Trichosurus vulpecula*, in lowland podocarp/mixed hardwood forest, Orongorongo Valley, New Zealand. *New Zealand journal of zoology*, *17*(4), 549-566.

Czarnecka, J., & Kitowski, I. (2010). Seed dispersal by the rook *Corvus frugilegus* L. In agricultural landscape- mechanisms and ecological importance. *Polish Journal of Ecology*, *58*(3), 511-523.

Czarnecka, J., Orłowski, G., & Karg, J. (2012). Endozoochorous dispersal of alien and native plants by two palearctic avian frugivores with special emphasis on invasive giant goldenrod *Solidago gigantea*. *Open Life Sciences, 7*(5), 895-901.

Da Silva, H. R., & De Britto-Pereira, M. C. (2006). How much fruit do fruit-eating frogs eat? An investigation on the diet of *Xenohyla truncata* (Lissamphibia: Anura: Hylidae). *Journal of Zoology*, *270*(4), 692-698.

Dalponte, J. C., & Tavares-Filho, J. A. (2004). Diet of the yellow armadillo, *Euphractus sexcinctus*, in south-central Brazil. *Edentata*, *2004*(6), 37-41.

Datta, A., & Rawat, G. S. (2003). Foraging patterns of sympatric hornbills during the nonbreeding season in Arunachal Pradesh, northeast India. *Biotropica*, *35*(2), 208-218.

Davis, N. E., Forsyth, D. M., & Coulson, G. (2010). Facilitative interactions between an exotic mammal and native and exotic plants: hog deer (*Axis porcinus*) as seed dispersers in south-eastern Australia. *Biological Invasions, 12*(5), 1079-1092.

de Azevedo, C. S., da Silva, M. C., Teixeira, T. P., Young, R. J., Garcia, Q. S., & Rodrigues, M. (2013). Effect of passage through the gut of Greater Rheas on the germination of seeds of plants of cerrado and caatinga grasslands. *Emu*, *113*, 177-182.

de Castro, E. R., Côrtes, M. C., Navarro, L., Galetti, M., & Morellato, L. P. C. (2012). Temporal variation in the abundance of two species of thrushes in relation to fruiting phenology in the Atlantic rainforest. *Emu-Austral Ornithology*, *112*(2), 137-148.

de Luna G., A., García-Morera, Y., & Link, A. (2016). Behavior and ecology of the white-footed tamarin (*Saguinus leucopus*) in a fragmented landscape of Colombia: Small bodied primates and seed dispersal in Neotropical forests. *Tropical Conservation Science, 9*(2), 788-808.

de Moura, A. C., Cavalcanti, L., Leite-Filho, E., Mesquita, D. O., & McConkey, K. R. (2015). Can green iguanas compensate for vanishing seed dispersers in the Atlantic forest fragments of north-east Brazil?. *Journal of Zoology*, *295*(3), 189-196.

de Vega, C., Arista, M., Ortiz, P. L., Herrera, C. M., & Talavera, S. (2011). Endozoochory by beetles: a novel seed dispersal mechanism. *Annals of Botany*, *107*(4), 629-637.

Dew, J. L. (2008). Spider monkeys as seed dispersers. In: C.J. Campbell (Ed.), *Spider monkeys: Behavior, ecology and evolution of the genus Ateles*, Cambridge University Press, Cambridge; pp. 155-182.

Dew, J. L., & Wright, P. (1998). Frugivory and Seed Dispersal by Four Species of Primates in Madagascar's Eastern Rain Forest 1. *Biotropica*, *30*(3), 425-437.

Dietz, J. M., Peres, C. A., & Pinder, L. (1997). Foraging ecology and use of space in wild golden lion tamarins (*Leontopithecus rosalia*). *American Journal of Primatology*, *41*(4), 289-305.

Dookia, S., & Jakher, G. R. (2007). Food and feeding habit of Indian Gazelle (*Gazella bennettii*), in the Thar Desert of Rajasthan. *Indian Forester, 133*(10), 1327-1340.

Downer, C. C. (2001). Observations on the diet and habitat of the mountain tapir (*Tapirus pinchaque*). *Journal of Zoology*, *254*(3), 279-291.

Dumbacher, J. P., & Beehler, B. M. (1996). More examples of fruiting trees visited predominantly by birds of paradise. *Emu*, *96* (2), 81-88.

Emura, N., Kawakami, K., Deguchi, T., & Sone, K. (2012). Potential role of frugivorous birds in the recovery process of forest vegetation after feral goat eradication in Mukojima Island, the Bonin Islands. *Journal of forest research*, *17*(4), 352-359.

Enders, M. S., & Vander Wall, S. B. (2012). Black bears *Ursus americanus* are effective seed dispersers, with a little help from their friends. *Oikos*, *121*(4), 589-596.

Entwistle, A., & Corp, N. (1997). The diet of *Pteropus voeltzkowi*, an endangered fruit bat endemic to Pemba Island, Tanzania. *African Journal of Ecology*, *35*(4), 351-360.

Fan, P., Ni, Q., Sun, G., Huang, B., & Jiang, X. (2009). Gibbons under seasonal stress: the diet of the black crested gibbon (*Nomascus concolor*) on Mt. Wuliang, Central Yunnan, China. *Primates, 50*(1), 37.

Farwig, N., Böhning-Gaese, K., & Bleher, B. (2006). Enhanced seed dispersal of *Prunus africana* in fragmented and disturbed forests?. *Oecologia*, *147*(2), 238-252.

Feer, F. (1995). Seed dispersal in African forest ruminants. *Journal of Tropical Ecology*, *11*(4), 683-689.

Fragoso, J. M., & Huffman, J. M. (2000). Seed-dispersal and seedling recruitment patterns by the last Neotropical megafaunal element in Amazonia, the tapir. *Journal of Tropical Ecology*, *16*(3), 369-385.

Fricke, E. C., Bender, J., Rehm, E. M., & Rogers, H. S. (2019). Functional outcomes of mutualistic network interactions: A community-scale study of frugivore gut passage on germination. *Journal of Ecology*, *107*(2), 757-767.

Galetti, M., Laps, R., & Pizo, M. A. (2000). Frugivory by toucans (Ramphastidae) at two altitudes in the Atlantic Forest of Brazil. *Biotropica*, *32*(4b), 842-850.

Garber, P. A., & Kitron, U. (1997). Seed swallowing in tamarins: evidence of a curative function or enhanced foraging efficiency?. *International Journal of Primatology*, *18*(4), 523-538.

Gatti, A., Bianchi, R., Rosa, C. R. X., & Mendes, S. L. (2006). Diet of two sympatric carnivores, Cerdocyon thous and Procyon cancrivorus, in a restinga area of Espirito Santo State, Brazil. *Journal of Tropical Ecology*, *22*(2), 227-230.

Gautier-Hion, A., Duplantier, J. M., Quris, R., Feer, F., Sourd, C., Decoux, J. P., ... & Moungazi, A. (1985). Fruit characters as a basis of fruit choice and seed dispersal in a tropical forest vertebrate community. *Oecologia, 65*(3), 324-337.

Giannini, N. P. (1999). La interacción de aves-murciélagos-plantas en el sistema de frugivoría y dispersión de semillas en San Javier, Tucumán, Argentina. PhD dissertation. *Universidad Nacional de Tucumán*.

González-Castro, A., Traveset, A., & Nogales, M. (2012). Seed dispersal interactions in the Mediterranean Region: contrasting patterns between islands and mainland. *Journal of Biogeography, 39*(11), 1938-1947.

Green, A. J., Jenkins, K. M., Bell, D., Morris, P. J., & Kingsford, R. T. (2008). The potential role of waterbirds in dispersing invertebrates and plants in arid Australia. *Freshwater Biology*, *53*(2), 380-392.

Guillotin, M., Dubost, G., & Sabatier, D. (1994). Food choice and food competition among the three major primate species of French Guiana. *Journal of Zoology, 233*(4), 551-579.

Guitian, J., Munilla, I., Guitian, P., & Lopez, B. (1994). Frugivory and seed dispersal by redwings *Turdus iliacus* in southwest Iceland. *Ecography, 17*(4), 314-320.

Gulraiz, T., Javid, A., Mahmood-Ul-Hassan, M., Hussain, S., Azmat, H., & Daud, S. (2016). Role of Indian flying fox *Pteropus giganteus* Brünnich, 1782 (Chiroptera: Pteropodidae) as a seed disperser in urban areas of Lahore, Pakistan. *Turkish Journal of Zoology*, *40*(3), 417-422.

Guzmán, A., Stevenson, P. (2008). Seed dispersal, habitat selection and movement patterns in the Amazonian tortoise, *Geochelone denticulata*. *Amphibia-Reptilia*, *29*(4), 463-472.

Hai, B. T., Chen, J., McConkey, K. R., & Dayananda, S. K. (2018). Gibbons (*Nomascus gabriellae*) provide key seed dispersal for the Pacific walnut (*Dracontomelon dao*), in Asia's lowland tropical forest. Acta *Oecologica, 88*, 71-79.

Hamann, A., & Curio, E. (1999). Interactions among frugivores and fleshy fruit trees in a Philippine submontane rainforest. *Conservation Biology*, *13*(4), 766-773.

Heithaus, E. R., Fleming, T. H., & Opler, P. A. (1975). Foraging patterns and resource utilization in seven species of bats in a seasonal tropical forest. *Ecology*, *56*(4), 841-854.

Heleno, R., Blake, S., Jaramillo, P., Traveset, A., Vargas, P., & Nogales, M. (2011). Frugivory and seed dispersal in the Galápagos: what is the state of the art? *Integrative Zoology*, *6*(2), 110-129.

Herrel, A., Vanhooydonck, B., Joachim, R., & Irschick, D. J. (2004). Frugivory in polychrotid lizards: effects of body size. *Oecologia*, *140*(1), 160-168.

Herrera, J. M., de Sá Teixeira, I., Rodríguez-Pérez, J., & Mira, A. (2016). Landscape structure shapes carnivore-mediated seed dispersal kernels. *Landscape ecology, 31*(4), 731-743.

Hervías-Parejo, S., Heleno, R., Rumeu, B., Guzmán, B., Vargas, P., Olesen, J. M., ... & Nogales, M. (2019). Small size does not restrain frugivory and seed dispersal across the evolutionary radiation of Galápagos lava lizards. *Current Zoology*, *65*(4), 353-361.

Heydon, M. J. (1994). The ecology and management of rain forest ungulates in Sabah, Malaysia: implications of forest disturbance. Institution of Tropical Biology. Final Report. University of Aberdeen.

Hickey, J. R., Flynn, R. W., Buskirk, S. W., Gerow, K. G., & Willson, M. F. (1999). An evaluation of a mammalian predator, *Martes americana*, as a disperser of seeds. *Oikos*, 499-508.

Holbrook, K. M., & Smith, T. B. (2000). Seed dispersal and movement patterns in two species of Ceratogymna hornbills in a West African tropical lowland forest. Oecologia, 125(2), 249-257.

Horn, A., Pachmann, G., & Poschlod, P. (2013). Can sheep replace indigenous antelope as seed dispersers in the Kalahari?. *Journal of Arid Environments, 91*, 69-78.

Jaroszewicz, B., Pirożnikow, E., & Sagehorn, R. (2009). Endozoochory by European bison (*Bison bonasus*) in Białowieża Primeval Forest across a management gradient. *Forest ecology and management*, *258*(1), 11-17.

Jaroszewicz, B., Pirożnikow, E., & Sondej, I. (2013). Endozoochory by the guild of ungulates in Europe's primeval forest. *Forest Ecology and Management*, *305*, 21-28.

Jerozolimski, A., Ribeiro, M. B. N., & Martins, M. (2009). Are tortoises important seed dispersers in Amazonian forests?. *Oecologia, 161*(3), 517-528.

Jordano, P., García, C., Godoy, J. A., & García-Castaño, J. L. (2007). Differential contribution of frugivores to complex seed dispersal patterns. *Proceedings of the National Academy of Sciences, 104*(9), 3278-3282.

Julliot, C. (1996). Fruit choice by red howler monkeys (*Alouatta seniculus*) in a tropical rain forest. *American Journal of Primatology*, *40*(3), 261-282.

Kaplin, B. A., & Moermond, T. C. (1998). Variation in seed handling by two species of forest monkeys in Rwanda. *American Journal of Primatology*, *45*(1), 83-101.

Karubian, J., Duraes, R., Storey, J. L., & Smith, T. B. (2012). Mating behavior drives seed dispersal by the long-wattled Umbrellabird *Cephalopterus penduliger. Biotropica, 44*(5), 689-698.

Kawakami, K., Mizusawa, L., & Higuchi, H. (2009). Re-established mutualism in a seed-dispersal system consisting of native and introduced birds and plants on the Bonin Islands, Japan. *Ecological Research*, *24*(4), 741-748.

Kays, R. W. (1999). Food preferences of kinkajous (*Potos flavus*). *Journal of Mammalogy*, *80*(2), 589-599.

Keuroghlian, A., & Eaton, D. P. (2008). Fruit availability and peccary frugivory in an isolated Atlantic forest fragment: effects on peccary ranging behavior and habitat use. *Biotropica*, *40*(1), 62-70.

Khamcha, D., Savini, T., Westcott, D. A., McKeown, A., Brockelman, W. Y., Chimchome, V., & Gale, G. A. (2014). Behavioral and social structure effects on seed dispersal curves of a forest-interior bulbul (Pycnonotidae) in a Tropical Evergreen Forest. *Biotropica*, *46*(3), 294-301.

King, P., Milicich L., and Burns, K.C. (2011). Body size determines rates of seed dispersal by giant king crickets. *Population ecology* 53.1: 73-80.

Kitamura, S., Yumoto, T., Poonswad, P., Chuailua, P., Plongmai, K., Maruhashi, T., & Noma, N. (2002). Interactions between fleshy fruits and frugivores in a tropical seasonal forest in Thailand. *Oecologia*, *133*(4), 559-572.

Kitamura, S., Yumoto, T., Poonswad, P., & Wohandee, P. (2007). Frugivory and seed dispersal by Asian elephants, *Elephas maximus*, in a moist evergreen forest of Thailand. *Journal of Tropical Ecology*, *23*(3), 373-376.

Kitamura, S., Thong-Aree, S., Madsri, S., & Poonswad, P. (2011). Characteristics of hornbill-dispersed fruits in lowland dipterocarp forests of southern Thailand. *The Raffles Bulletin of Zoology*, *24*, 137-147.

Kleyheeg, E., & van Leeuwen, C. H. (2015). Regurgitation by waterfowl: An overlooked mechanism for long-distance dispersal of wetland plant seeds. *Aquatic Botany*, *127*, 1-5.

Knogge, C., & Heymann, E. W. (2003). Seed dispersal by sympatric tamarins, *Saguinus mystax* and *Saguinus fuscicollis*: diversity and characteristics of plant species. *Folia Primatologica*, *74*(1), 33-47.

Koike, S., & Masaki, T. (2019). Characteristics of fruits consumed by mammalian frugivores in Japanese temperate forest. *Ecological Research*, *34*(2), 246-254.

Korine, C., Izhaki, I., & Arad, Z. (1999). Is the Egyptian fruit-bat *Rousettus aegyptiacus* a pest in Israel? An analysis of the bat's diet and implications for its conservation. *Biological Conservation*, *88*(3), 301-306.

Kubitzki, K., & Ziburski, A. (1994). Seed dispersal in flood plain forests of Amazonia. *Biotropica*, 30-43.

Kunz, B. K., & Linsenmair, K. E. (2008). The role of the olive baboon (*Papio anubis*, Cercopithecidae) as seed disperser in a savanna-forest mosaic of West Africa. *Journal of Tropical Ecology*, *24*(3), 235-246.

Lahann, P. (2007). Feeding ecology and seed dispersal of sympatric cheirogaleid lemurs (*Microcebus murinus, Cheirogaleus medius, Cheirogaleus major*) in the littoral rainforest of south-east Madagascar. *Journal of Zoology*, *271*(1), 88-98.

Leiner, N. O., & Silva, W. R. (2007). Seasonal variation in the diet of the Brazilian slender opossum (*Marmosops paulensis*) in a montane Atlantic forest area, southeastern Brazil. *Journal of Mammalogy*, *88*(1), 158-164.

Lenz, J., Fiedler, W., Caprano, T., Friedrichs, W., Gaese, B. H., Wikelski, M., & Böhning-Gaese, K. (2011). Seed-dispersal distributions by trumpeter hornbills in fragmented landscapes. *Proceedings of the Royal Society B: Biological Sciences, 278*(1716), 2257-2264.

Lessa, L. G., & da Costa, F. N. (2010). Diet and seed dispersal by five marsupials (Didelphimorphia: Didelphidae) in a Brazilian cerrado reserve. *Mammalian Biology*, *75*(1), 10-16.

Lieberman, D., Lieberman, M., & Martin, C. (1987). Notes on seeds in elephant dung from Bia National Park, Ghana. *Biotropica*, 365-369.

Lindell, C. A., Reid, J. L., & Cole, R. J. (2013). Planting design effects on avian seed dispersers in a tropical forest restoration experiment. *Restoration Ecology*, *21*(4), 515-522.

Link, A., & Di Fiore, A. (2006). Seed dispersal by spider monkeys and its importance in the maintenance of neotropical rain-forest diversity. *Journal of Tropical Ecology*, *22*(3), 235-246.

Link, A., Galvis, N., Marquez, M., Guerrero, J., Solano, C., & Stevenson, P. R. (2012). Diet of the critically endangered brown spider monkey (*Ateles hybridus*) in an inter-Andean lowland rainforest in Colombia. *American Journal of Primatology*, *74*(12), 1097-1105.

Liu, H., Platt, S. G., & Borg, C. K. (2004). Seed dispersal by the Florida box turtle (*Terrapene carolina bauri*) in pine rockland forests of the lower Florida Keys, United States. *Oecologia*, *138*(4), 539-546.

Lopez-Darias, M., & Nogales, M. (2008). Effects of the invasive Barbary ground squirrel (*Atlantoxerus getulus*) on seed dispersal systems of insular xeric environments. *Journal of Arid Environments*, *72*(6), 926-939.

Lucas, C. M. (2008). Within flood season variation in fruit consumption and seed dispersal by two characin fishes of the Amazon. *Biotropica*, *40*(5), 581-589.

Lucas, P. W., & Corlett, R. T. (1998). Seed dispersal by long-tailed macaques. *American Journal of Primatology*, *45*(1), 29-44.

Mancilla-Leytón, J. M., González-Redondo, P., & Vicente, A. M. (2013). Effects of rabbit gut passage on seed retrieval and germination of three shrub species. *Basic and Applied Ecology*, *14*(7), 585-592.

Mannheimer, S., Bevilacqua, G., Caramaschi, E. P., & Scarano, F. R. (2003). Evidence for seed dispersal by the catfish *Auchenipterichthys longimanus* in an Amazonian lake. *Journal of Tropical Ecology*, *19*(2), 215-218.

Martins, M. M. (2008). Fruit diet of *Alouatta guariba* and *Brachyteles arachnoides* in Southeastern Brazil: comparison of fruit type, color, and seed size. *Primates*, *49*(1), 1-8.

McConkey, K. R. (2000). Primary seed shadow generated by gibbons in the rain forests of Barito Ulu, central Borneo. *American Journal of Primatology*, *52*(1), 13-29.

McConkey, K. R., & Drake, D. R. (2006). Flying foxes cease to function as seed dispersers long before they become rare. *Ecology*, *87*(2), 271-276.

McConkey, K. I. M., & Galetti, M. (1999). Seed dispersal by the sun bear *Helarctos malayanus* in Central Borneo. *Journal of Tropical Ecology*, *15*(2), 237-241.

McConkey, K. R., Meehan, H. J., & Drake, D. R. (2004). Seed dispersal by Pacific pigeons (*Ducula pacifica*) in Tonga, western Polynesia. *Emu-Austral Ornithology*, *104*(4), 369-376.

McEwan, W. M. (1978). The food of the New Zealand pigeon (*Hemiphaga novaeseelandiae novaeseelandiae*). *New Zealand Journal of Ecology*, 99-108.

Milton, S. J., & Dean, W. R. J. (2001). Seeds dispersed in dung of insectivores and herbivores in semi-arid southern Africa. *Journal of Arid Environments, 47*(4), 465-483.

Moll, D., & Jansen, K. P. (1995). Evidence for a role in seed dispersal by two tropical herbivorous turtles. *Biotropica*, 121-127.

Montaño-Centellas, F. A. (2012). Are males and females of Yungas Manakin (*Chiroxiphia boliviana*) ecologically redundant as seed dispersers. *Ornitologia Neotropical*, *23*, 185-192.

Moses, K. L., & Semple, S. (2011). Primary seed dispersal by the black-and-white ruffed lemur (*Varecia variegata*) in the Manombo forest, South-east Madagascar. *Journal of Tropical Ecology*, *27*(5), 529-538.

Moura, A. C., & McConkey, K. R. (2007). The capuchin, the howler, and the caatinga: seed dispersal by monkeys in a threatened Brazilian forest. *American Journal of Primatology: Official Journal of the American Society of Primatologists*, *69*(2), 220-226.

Mueller, M. H., & van der Valk, A. G. (2002). The potential role of ducks in wetland seed dispersal. *Wetlands*, *22*(1), 170-178.

Murdoch, J. D., Buyandelger, S., & Cypher, B. L. (2009). Patterns of seed occurrence in corsac and red fox diets in Mongolia. *Journal of Arid Environments*, *73*(3), 381-384.

Murray, K. G. (1988). Avian seed dispersal of three neotropical gap-dependent plants. *Ecological monographs*, *58*(4), 271-298.

Nago, R., Kobayashi, S., Kinjo, T., Ogimi, K., Sinjo, A., Namisato, S., ... & Izawa, M. (2019). Seed feeding behavior of *Diplothrix legata* (Muridae: Rodentia): Effects on germination of five plants in Okinawajima Island, Japan. *Mammal Study*, *44*(2), 129-134.

Nakamoto, A., Kinjo, K., & Izawa, M. (2009). The role of Orii's flying-fox (*Pteropus dasymallus inopinatus*) as a pollinator and a seed disperser on Okinawa-Jima Island, the Ryukyu Archipelago, Japan. *Ecological Research*, *24*(2), 405-414.

Naranjo, E. J. (2009). Ecology and conservation of Baird's tapir in Mexico. *Tropical Conservation Science*, *2*(2), 140-158.

Nield, A. P., Nathan, R., Enright, N. J., Ladd, P. G., & Perry, G. L. (2020). The spatial complexity of seed movement: Animal-generated seed dispersal patterns in fragmented landscapes revealed by animal movement models. *Journal of Ecology, 108*(2), 687-701.

Nishi, H., & Tsuyuzaki, S. (2004). Seed dispersal and seedling establishment of Rhus trichocarpa promoted by a crow (*Corvus macrorhynchos*) on a volcano in Japan. *Ecography*, *27*(3), 311-322.

Nogales, M., & Hernández, E. C. (1994). Interinsular variations in the spring and summer diet of the Raven *Corvus corax* in the Canary Islands. *Ibis*, *136*(4), 441-447.

Noma, N., & Yumoto, T. (1997). Fruiting phenology of animal-dispersed plants in response to winter migration of frugivores in a warm temperate forest on Yakushima Island, Japan. *Ecological Research, 12*(2), 119-129.

Norconk, M. A., Grafton, B. W., & Conklin-Brittain, N. L. (1998). Seed dispersal by Neotropical seed predators. *American Journal of Primatology*, *45*(1), 103-126.

Nyhagen, D. F., Turnbull, S. D., Olesen, J. M., & Jones, C. G. (2005). An investigation into the role of the Mauritian flying fox, *Pteropus niger*, in forest regeneration. *Biological Conservation*, *122*(3), 491-497.

Oleksy, R. (2015). The impact of the Mauritius fruit bat (*Pteropus niger*) on commercial fruit farms and possible mitigation measures. Final report, The Rufford Foundation.

Oliveira, A. C. M., & Ferrari, S. F. (2000). Seed dispersal by black-handed tamarins, *Saguinus midas niger* (Callitrichinae, Primates): implications for the regeneration of degraded forest habitats in eastern Amazonia. *Journal of Tropical Ecology*, *16*(5), 709-716.

Oliveira-Silva, L. R. B., Campêlo, A. C., Lima, I. M. S., Araújo, A. C. L., Bezerra, B. M., & Souza-Alves, J. P. (2018). Can a non-native primate be a potential seed disperser? A case study on *Saimiri sciureus* in Pernambuco state, Brazil. *Folia Primatologica*, *89*(2), 138-149.

Orłowski, G., & Czarnecka, J. (2012). Re-evaluation of the role of the grey partridge *Perdix perdix* as a disperser of arable weed seeds. *Journal of Ornithology*, *154*(1), 139-144.

Padgett, D. J., Carboni, J. J., & Schepis, D. J. (2010). The dietary composition of *Chrysemys picta picta* (eastern painted turtles) with special reference to the seeds of aquatic macrophytes. *Northeastern* Naturalist, 17(2), 305-312.

Palma, A. C., & Stevenson, P. R. (2010). Dispersión de semillas por monos araña en la estación biológica Cocha Cashu, Perú. In Pereira-Bengoa, V., Stevenson, P. R., Bueno, M. & Nassar-Montoya, F. (eds) Primatología en Colombia: Avances al principio del milenio, pp. 19-35. Fundación Universitaria San Martín. Bogotá.

Palmer, C., Price, O., & Bach, C. (2000). Foraging ecology of the black flying fox (*Pteropus alecto*) in the seasonal tropics of the Northern Territory, Australia. Wildlife Research, 27(2), 169-178.

Panter, C. J., & Dolman, P. M. (2012). Mammalian herbivores as potential seed dispersal vectors in ancient woodland fragments. *Wildlife Biology*, *18*(3), 292-303.

Pendje, G. (1994). Fruit consumption and seed dispersal by the African civet *Civettictis civetta* in Mayombe, Zaire. *Revue D Ecologie-La Terre Et La Vie*, *49*(2), 107-116.

Pérez-Mellado, V., & Traveset, A. (1999). Relationships between plants and Mediterranean lizards. *Natura Croatica: Periodicum Musei Historiae Naturalis Croatici*, *8*(3), 275-285.

Phiphatsuwannachai, S., Westcott, D. A., McKeown, A., & Savini, T. (2018). Inter-group variability in seed dispersal by white-handed gibbons in mosaic forest. *Biotropica*, *50*(1), 106-115.

Piazzon, M., Larrinaga, A. R., Rodríguez-Pérez, J., Latorre, L., Navarro, L., & Santamaría, L. (2012). Seed dispersal by lizards on a continental-shelf island: predicting interspecific variation in seed rain based on plant distribution and lizard movement patterns. *Journal of Biogeography*, *39*(11), 1984-1995.

Picot, M., Jenkins, R.K.B., Ramilijaona, O., Racey, P.A. & Carrière, S.M. (2007) The feeding ecology of *Eidolon dupreanum* (Pteropodidae) in eastern Madagascar. *African Journal of Ecology*, *45*, 645-650.

Pizo, M. A. (2004). Frugivory and habitat use by fruit-eating birds in a fragmented landscape of southeast Brazil. *Ornitologia Neotropical*, *15*(1), 117-126.

Platt, S. G., Manning, P. R., & Rainwater, T. R. (2014). Consumption of desert gourds by collared peccary suggests the fruit is not an ecological anachronism. *The Southwestern Naturalist*, *59*(1), 141-144.

Platt, S. G., Berezin, A. R., Miller, D. J., & Rainwater, T. R. (2016). A dietary study of the rough-footed mud turtle (*Kinosternon hirtipes*) in Texas, USA. *Herpetological Conservation and Biology*, *11*(1), 142-149.

Poulsen, J. R., Clark, C. J., & Smith, T. B. (2001). Seed dispersal by a diurnal primate community in the Dja Reserve, Cameroon. *Journal of Tropical Ecology*, *17*(6), 787-808.

Ragusa-Netto, J. (2006). Abundance and frugivory of the toco toucan (*Ramphastos toco*) in a gallery forest in Brazil's Southern Pantanal. *Brazilian Journal of Biology*, *66*(1A), 133-142.

Ramananjato, V., Rakotomalala, Z., Park, D. S., DeSisto, C. M., Raoelinjanakolona, N. N., Guthrie, N. K., ... & Razafindratsima, O. H. 2020. The role of nocturnal omnivorous lemurs as seed dispersers in Malagasy rain forests. *Biotropica*. DOI: 10.1111/btp.12789

Ramirez, M. A., Galvis N. F., Vargas S., Leon J. J., Cifuentes E., Stevenson P. R. 2014. Seed dispersal by woolly monkeys in Cueva de Los Guacharos National Park (Colombia): an amazonian primate dispersing mountain plants. In Krzton A, Gursky S, Grow NB (eds); pp. 103-114. High Altitude Primates. Springer Developments in Primatology.

Raulings, E., Morris, K. A. Y., Thompson, R., & Nally, R. M. (2011). Do birds of a feather disperse plants together?. *Freshwater Biology*, *56*(7), 1390-1402.

Razafindratsima, O.H. & Martinez, B.T. (2012) Seed dispersal by red-ruffed lemurs: seed size, viability, and beneficial effect on seedling growth. *Ecotropica, 18*, 15-25.

Razafindratsima, O. H., Jones, T. A., & Dunham, A. E. (2014). Patterns of movement and seed dispersal by three lemur species. *American Journal of Primatology*, *76*(1), 84-96.

Regnier, E., Harrison, S. K., Liu, J., Schmoll, J. T., Edwards, C. A., Arancon, N., & Holloman, C. (2008). Impact of an exotic earthworm on seed dispersal of an indigenous US weed. *Journal of Applied Ecology*, *45*(6), 1621-1629.

Reid, S. & Armesto, J.J. (2011) Interaction dynamics of avian frugivores and plants in a Chilean Mediterranean shrubland. *Journal of Arid Environments, 75*, 221-230.

Richards, G. C. (1990). The spectacled flying-fox, *Pteropus conspicillat*us (Chiroptera: Pteropodidae), in north Queensland. 2. Diet, seed dispersal and feeding ecology. *Australian Mammalogy*, *13*, 25-31.

Riehl, C., & Adelson, G. S. (2008). Seasonal insectivory by Black-headed Trogons, a tropical dry forest frugivore. *Journal of Field Ornithology*, *79*(4), 371-380.

Rosado, B. B., & Linares, H. D. (2017). Estrategia alimentaria, de *Pteroglossus castanotis*, Ramphastidae. *Biodiversidad Amazónica 5*, *1*(1).

Rosalino, L. M., Rosa, S., & Santos-Reis, M. (2010). The role of carnivores as Mediterranean seed dispersers. In *Annales Zoologici Fennici* (Vol. 47, No. 3, pp. 195-205). Finnish Zoological and Botanical Publishing Board.

Rosas, C. A., Engle, D. M., Shaw, J. H., & Palmer, M. W. (2008). Seed dispersal by *Bison bison* in a tallgrass prairie. *Journal of Vegetation Science*, *19*(6), 769-778.

Ruggera, R. A., Gomez, M. D., & Blendinger, P. G. (2014). Frugivory and seed dispersal role of the Yellow-striped Brush-Finch (*Atlapetes citrinellus*), an endemic emberizid of Argentina. *Emu-Austral Ornithology, 114*(4), 343-351.

Russo, S. E., Portnoy, S., & Augspurger, C. K. (2006). Incorporating animal behavior into seed dispersal models: implications for seed shadows. *Ecology, 87*(12), 3160-3174.

Sánchez, M. S., Giannini, N. P., & Barquez, R. M. (2012). Bat frugivory in two subtropical rain forests of Northern Argentina: testing hypotheses of fruit selection in the Neotropics. *Mammalian Biology, 77*(1), 22-31.

Sankamethawee, W., Pierce, A. J., Gale, G. A., & Hardesty, B. D. (2011). Plant-frugivore interactions in an intact tropical forest in northeast Thailand. *Integrative Zoology, 6*(3), 195-212.

Santamaría, M., & Franco, A. M. (1994). Historia natural del paujil *Mitu salvini* y densidades poblacionales de los crácidos en el Parque Nacional Natural Tinigua-Amazonia colombiana. Wildlife Conservation Society, Bogotá, Colombia.

Sato, H. (2012). Frugivory and seed dispersal by brown lemurs in a Malagasy tropical dry forest. *Biotropica*, *44*(4), 479-488.

Sato, H. (2018). Predictions of seed shadows generated by common brown lemurs (*Eulemur fulvus*) and their relationship to seasonal behavioral strategies. *International Journal of Primatology, 39*(3), 377-396.

Schaumann, F., & Heinken, T. (2002). Endozoochorous seed dispersal by martens (*Martes foina*, M. *martes*) in two woodland habitats. *Flora-Morphology, Distribution, Functional Ecology of Plants*, *197*(5), 370-378.

Schupp, E. W., Heaton, H. J., & Gomez, J. M. (1997). Lagomorphs and the dispersal of seeds into communities dominated by exotic annual weeds. *The Great Basin Naturalist, 57*(3), 253-258.

Sethi, P., & Howe, H. F. (2012). Fruit removal by hornbills in a semi-evergreen forest of the Indian Eastern Himalaya. *Journal of Tropical Ecology, 28*(6), 531-541.

Shiponeni, N. N., & Milton, S. J. (2006). Seed dispersal in the dung of large herbivores: implications for restoration of Renosterveld shrubland old fields. *Biodiversity & Conservation*, *15*(10), 3161-3175.

Silva, S. I., Bozinovic, F., & Jaksic, F. M. (2005). Frugivory and seed dispersal by foxes in relation to mammalian prey abundance in a semiarid thornscrub. *Austral Ecology*, *30*(7), 739-746.

Silveira, M., Trevelin, L., Port-Carvalho, M., Godoi, S., Mandetta, E. N., & Cruz-Neto, A. P. (2011). Frugivory by phyllostomid bats (Mammalia: Chiroptera) in a restored area in Southeast Brazil. *Acta Oecologica, 37*(1), 31-36.

Slater, K., & Du Toit, J. T. (2002). Seed dispersal by chacma baboons and syntopic ungulates in southern African savannas. *South African Journal of Wildlife Research-24-month delayed open access*, *32*(1), 75-79.

Sloan, K. N., Buhlmann, K. A., & Lovich, J. E. (1996). Stomach contents of commercially harvested adult alligator snapping turtles, *Macroclemys temminckii*.

Spotswood, E. N., Meyer, J. Y., & Bartolome, J. W. (2012). An invasive tree alters the structure of seed dispersal networks between birds and plants in French Polynesia. *Journal of Biogeography*, *39*(11), 2007-2020.

Sridhara, S., McConkey, K., Prasad, S., & Corlett, R. T. (2016). Frugivory and seed dispersal by large herbivores of Asia. In *The ecology of large herbivores in South and Southeast Asia* (pp. 121-150). Springer, Dordrecht.

Stevenson, P. R., Cardona, L. M., Acosta-Rojas, D. C., Henao-Diaz, F., & Cárdenas, S. (2017). Diet of oilbirds (*Steatornis caripensis*) in Cueva de los Guácharos National Park (Colombia): temporal variation in fruit consumption, dispersal, and seed morphology. *Ornitología Neotropical, 28*, 295-307.

Stocker, G. C., & Irvine, A. K. (1983). Seed dispersal by cassowaries (*Casuarius casuarius*) in North Queensland's rainforests. *Biotropica, 15*(3), 170-176.

Subrata, S. A., & Syahbudin, A. (2016). Common palm civet as a potential seed disperser of important plant species in Java. In *AIP Conference Proceedings* (Vol. 1744, No. 1, p. 020053). AIP Publishing LLC.

Sun, C., Moermond, T. C., & Givnish, T. J. (1997). Nutritional determinants of diet in three turacos in a tropical montane forest. *The Auk*, *114*(2), 200-211.

Sung, Y. H., Hau, B. C., & Karraker, N. E. (2016). Diet of the endangered big-headed turtle *Platysternon megacephalum*. *PeerJ, 4*, e2784.

Takahashi, K., Shiota, T., Tamatani, H., Koyama, M., & Washitani, I. (2008). Seasonal variation in fleshy fruit use and seed dispersal by the Japanese black bear (*Ursus thibetanus japonicus*). *Ecological Research*, *23*(2), 471-478.

Tarszisz, E., Tomlinson, S., Harrison, M. E., Morrogh-Bernard, H. C., & Munn, A. J. (2018). Gardeners of the forest: effects of seed handling and ingestion by orangutans on germination success of peat forest plants. *Biological Journal of the Linnean Society, 123*(1), 125-134.

Tellería, L. J., Blázquez, M., De La Hera, I., & Pérez-Tris, J. (2013). Migratory and resident Blackcaps *Sylvia atricapilla* wintering in southern Spain show no resource partitioning. *Ibis*, *155*(4), 750-761.

Thiry, V., Bhasin, O., Stark, D. J., Beudels-Jamar, R. C., Drubbel, R. V., Nathan, S. K., ... & Vercauteren, M. (2019). Seed dispersal by proboscis monkeys: the case of *Nauclea* spp. *Primates*, *60*(5), 449-457.

Traveset, A. (1990). *Ctenosaura similis* Gray (Iguanidae) as a seed disperser in a Central American deciduous forest. *American Midland Naturalist*, 402-404.

Trolliet, F., Serckx, A., Forget, P. M., Beudels-Jamar, R. C., Huynen, M. C., & Hambuckers, A. (2016). Ecosystem services provided by a large endangered primate in a forest-savanna mosaic landscape. *Biological conservation*, *203*, 55-66.

Tsuji, Y., Sato, K., & Sato, Y. (2011a). The role of Japanese macaques (*Macaca fuscata*) as endozoochorous seed dispersers on Kinkazan Island, northern Japan. *Mammalian Biology*, *76*(5), 525-533.

Tsuji, Y., Tatewaki, T., & Kanda, E. (2011b). Endozoochorous seed dispersal by sympatric mustelids, *Martes melampus* and *Mustela itatsi*, in western Tokyo, central Japan. *Mammalian Biology*, *76*(5), 628-633.

Tsuji, Y., Hanya, G., & Grueter, C. C. (2013). Feeding strategies of primates in temperate and alpine forests: comparison of Asian macaques and colobines. *Primates, 54*(3), 201-215.

Tsuji, Y., Ningsih, J. I. D. P., Kitamura, S., Widayati, K. A., & Suryobroto, B. (2017). Neglected seed dispersers: endozoochory by Javan lutungs (*Trachypithecus auratus*) in Indonesia. *Biotropica*, *49*(4), 539-545.

Türke, M., & Weisser, W. W. (2013). Species, diaspore volume and body mass matter in gastropod seed feeding behavior. *PloS one*, *8*(7).

Tutin, C. E., Williamson, E. A., Rogers, M. E., & Fernandez, M. (1991). A case study of a plant-animal relationship: *Cola lizae* and lowland gorillas in the Lopé Reserve, Gabon. *Journal of Tropical Ecology*, *7*(2), 181-199.

Valido, A., & Nogales, M. (1994). Frugivory and seed dispersal by the lizard *Gallotia galloti* (Lacertidae) in a xeric habitat of the Canary Islands. *Oikos*, 403-411.

Varela, O., & Bucher, E. H. (2006). Passage time, viability, and germination of seeds ingested by foxes. *Journal of Arid Environments*, *67*(4), 566-578.

Varela, O., Cormenzana-Méndez, A., Krapovickas, L., & Bucher, E. H. (2008). Seasonal diet of the Pampas fox (*Lycalopex gymnocercus*) in the Chaco dry woodland, northwestern Argentina. *Journal of Mammalogy*, *89*(4), 1012-1019.

Varela, R. O., & Bucher, E. H. (2002a). Seed dispersal by *Chelonoidis chilensis* in the Chaco dry woodland of Argentina. *Journal of Herpetology*, *36*(1), 137-140.

Varela, R. O., & Bucher, E. H. (2002b). The lizard *Teius teyou* (Squamata: Teiidae) as a legitimate seed disperser in the dry Chaco forest of Argentina. *Studies on Neotropical Fauna and Environment*, *37*(2), 115-117.

Vellend, M., Myers, J. A., Gardescu, S., & Marks, P. L. (2003). Dispersal of *Trillium* seeds by deer: implications for long-distance migration of forest herbs. *Ecology, 84*(4), 1067-1072.

Waibel, A., Griffiths, C. J., Zuël, N., Schmid, B., & Albrecht, M. (2013). Does a giant tortoise taxon substitute enhance seed germination of exotic fleshy-fruited plants?. *Journal of Plant Ecology*, *6*(1), 57-63.

Webala, P. W., Musila, S., & Makau, R. (2014). Roost occupancy, roost site selection and diet of straw-coloured fruit bats (Pteropodidae: *Eidolon helvum*) in western Kenya: the need for continued public education. *Acta Chiropterologica*, *16*(1), 85-94.

Wehncke, E. V., Valdez, C. N., & Domínguez, C. A. (2004). Seed dispersal and defecation patterns of *Cebus capucinus* and *Alouatta palliata*: consequences for seed dispersal effectiveness. *Journal of Tropical Ecology*, *20*(5), 535-543.

Weinkam, T. J., Janos, G. A., & Brown, D. R. (2017). Habitat use and foraging behavior of Eastern Bluebirds (*Sialia sialis*) in relation to winter weather. *Northeastern Naturalist*, *24*(sp7), B1-B18.

Weir, J. E., & Corlett, R. T. (2007). How far do birds disperse seeds in the degraded tropical landscape of Hong Kong, China?. Landscape Ecology, 22(1), 131-140.

Westcott, D. A., Bentrupperbäumer, J., Bradford, M. G., & McKeown, A. (2005). Incorporating patterns of disperser behaviour into models of seed dispersal and its effects on estimated dispersal curves. Oecologia, 146(1), 57-67.

Wheelwright, N. T., Haber, W. A., Murray, K. G., & Guindon, C. (1984). Tropical fruit-eating birds and their food plants: a survey of a Costa Rican lower montane forest. *Biotropica*, 173-192.

Whitaker, A. H. (1987). The roles of lizards in New Zealand plant reproductive strategies. *New Zealand Journal of Botany*, *25*(2), 315-328.

Whitney, K. D., Fogiel, M. K., Lamperti, A. M., Holbrook, K. M., Stauffer, D. J., Hardesty, B. D., ... & Smith, T. B. (1998). Seed dispersal by *Ceratogymna* hornbills in the Dja Reserve, Cameroon. *Journal of Tropical Ecology*, *14*(3), 351-371.

Williams, P.A. & Karl, B.J. (1996) Fleshy fruits of indigenous and adventive plants in the diet of birds in forest remnants, Nelson, New Zealand. *New Zealand Journal of Ecology, 20*, 127-145.

Williams, S. C., Ward, J. S., & Ramakrishnan, U. (2008). Endozoochory by white-tailed deer (*Odocoileus virginianus*) across a suburban/woodland interface. *Forest Ecology and Management*, *255*(3-4), 940-947.

Willson, M. F., Sabag, C., Figueroa, J., Armesto, J. J., & Caviedes, M. (1996). Seed dispersal by lizards in Chilean rainforest. *Revista Chilena de Historia Natural*, *69*(3), 339-342.

Witmer, M. C. (1996). Annual diet of Cedar Waxwings based on US Biological Survey records (1885-1950) compared to diet of American Robins: contrasts in dietary patterns and natural history. *The Auk*, *113*(2), 414-430.

Wood. C. (1924). The Polynesian fruit pigeon, *Globicera pacifica*. Its food and digestive apparatus. *Auk 41*, 433-438.

Wotton, D. M. (2002). Effectiveness of the common gecko (*Hoplodactylus maculatus*) as a seed disperser on Mana Island, New Zealand. *New Zealand Journal of Botany*, *40*(4), 639-647.

Wotton, D. M., & Kelly, D. (2012). Do larger frugivores move seeds further? Body size, seed dispersal distance, and a case study of a large, sedentary pigeon. *Journal of Biogeography*, *39*(11), 1973-1983.

Wyman, T. E., & Kelly, D. (2017). Quantifying seed dispersal by birds and possums in a lowland New Zealand forest. *New Zealand Journal of Ecology*, *41*(1), 47-55.

Yamashiro, A., & Yamashiro, T. (2006). Seed Dispersal by Kerama Deer (*Cervus nippon keramae*) on Aka Island, the Ryukyu Archipelago, Japan. *Biotropica*, *38*(3), 405-413.

Young, L. M., Kelly, D., & Nelson, X. J. (2012). Alpine flora may depend on declining frugivorous parrot for seed dispersal. *Biological Conservation*, *147*(1), 133-142.

Zapata-Mesa, N., Montoya-Bustamante, S., & Murillo-García, O. E. (2017). Temporal variation in bat-fruit interactions: Foraging strategies influence network structure over time. *Acta Oecologica*, *85*, 9-17.

Zárate, D. A., Andresen, E., Estrada, A., & Serio-silva, J. C. (2014). Black howler monkey (*Alouatta pigra*) activity, foraging and seed dispersal patterns in shaded cocoa plantations versus rainforest in southern Mexico. *American Journal of Primatology, 76*(9), 890-899.

Zedler, P. H., & Black, C. (1992). Seed dispersal by a generalized herbivore: rabbits as dispersal vectors in a semiarid California vernal pool landscape. *American Midland Naturalist*, 1-10.

Zhou, Y. B., Zhang, L., Kaneko, Y., Newman, C., & Wang, X. M. (2008a). Frugivory and seed dispersal by a small carnivore, the Chinese ferret-badger, *Melogale moschata*, in a fragmented subtropical forest of central China. *Forest Ecology and Management*, *255*(5-6), 1595-1603.

Zhou, Y. B., Slade, E., Newman, C., Wang, X. M., & Zhang, S. Y. (2008b). Frugivory and seed dispersal by the yellow-throated marten, *Martes flavigula*, in a subtropical forest of China. *Journal of Tropical Ecology*, *24*(2), 219-223.

Zhou, Y., Zhang, J., Slade, E., Zhang, L., Palomares, F., Chen, J., ... & Zhang, S. (2008). Dietary shifts in relation to fruit availability among masked palm civets (*Paguma larvata*) in central China. *Journal of Mammalogy, 89*(2), 435-447.
